# CD36-BATF2\MYB Axis Predicts Anti-PD-1 Immunotherapy Response in Gastric Cancer

**DOI:** 10.7150/ijbs.87635

**Published:** 2023-08-21

**Authors:** Qiuyu Jiang, Zhixue Chen, Fansheng Meng, Hao Zhang, He Chen, Jindan Xue, Xizhong Shen, Tianshu Liu, Ling Dong, Si Zhang, Ruyi Xue

**Affiliations:** 1 Department of Gastroenterology and Hepatology, Shanghai Institute of Liver Diseases, Zhongshan Hospital, Fudan University, Shanghai, 200032, China.; 2 Liver Cancer Institute, Zhongshan Hospital, Fudan University, Shanghai, 200032, China.; 3 Department of Oncology, Minhang Hospital, Fudan University, China.; 4 Key Laboratory of Whole-Period Monitoring and Precise Intervention of Digestive Cancer (SMHC), Minhang Hospital & AHS, Fudan University, China.; 5 School of Medicine, Anhui University of Science and Technology, Anhui, 232000, China.; 6 Department of Medical Oncology, Zhongshan Hospital, Fudan University, Shanghai 200032, China.; 7 NHC Key Laboratory of Glycoconjugate Research, Department of Biochemistry and Molecular Biology, School of Basic Medical Sciences, Fudan University, Shanghai, 200032, China.; 8 Shanghai Baoshan District Wusong Central Hospital (Zhongshan Hospital Wusong Branch, Fudan University), Shanghai 200940, China.

**Keywords:** Gastric cancer, Immune cell related genes, Prognosis, Risk signature, Activated CD4+ memory T cells, Immunotherapy

## Abstract

Despite the utilization of anti-PD-1 therapy in gastric cancer (GC), the absence of a reliable predictive biomarker continues to pose a challenge. In this study, we utilized bioinformatic analysis and immunohistochemistry to develop a prediction model for activated CD4+ memory T cells, considering both mRNA and protein levels. An elevation of activated CD4+ memory T cells in GC was noted, which exhibited a strong association with the patients' overall survival. By utilizing WGCNA and DEG analysis, we discovered that BATF2, MYB, and CD36 are genes that exhibit differential expression and are linked to activated CD4+ memory T cells. Afterwards, a forecast model was built utilizing Stepwise regression and immunohistochemistry relying on the three genes. The model's high-risk score showed significant associations with a suppressive immune microenvironment. Moreover, our model exhibited encouraging prognostic value and superior performance in predicting response to immune checkpoint blockade therapy compared with the conventional CD8+PD-L1 model. In terms of mechanism, CD36 could function as a receptor upstream that identifies Helicobacter pylori and fatty acids. This recognition then results in the reduction of the BATF2-MYB protein complex and subsequent alterations in the transcription of genes associated with classical T cell activation. As a result, the activation state of CD4+ memory T cells is ultimately suppressed. The CD36-BATF2/MYB signature serves as a robust predictor of anti-PD-1 immunotherapy response in GC.

## Introduction

Globally, gastric cancer (GC) is the fifth most common cancer and the third leading cause of cancer-related deaths^1^. Due to the low rate of early detection, most patients (70%) are diagnosed with advanced-stage illness, resulting in the missed opportunity for optimal surgical intervention^2^. Hence, the general outlook for GC is bleak, with a less than 20% survival rate over a span of 5 years^3^. After undergoing several rounds of treatment, immune checkpoint inhibitors (ICIs) specifically targeting the PD-1/PD-L1 axis have shown great effectiveness in treating advanced gastric cancer that is unresponsive to chemotherapy. Currently, in the United States, pembrolizumab, which is an Anti-PD-1 agent, has received approval for treating patients suffering advanced gastric cancer with high PD-L1 expression. Nevertheless, various experiments involving pembrolizumab or nivolumab have indicated relatively modest rates of response (ranging from 10% to 26%) in gastric cancer during the salvage phase, without the presence of any specific biomarker or PD-L1 positivity^4^. The economic burden and significant side effects associated with anti-PD-1 therapy make it unsuitable for indiscriminate administration. Therefore, the judicious selection of appropriate candidates is crucial when considering the application of anti-PD-1 treatment. Hence, it is imperative to discover novel biomarkers that can assist in identifying patients who are eligible for anti-PD-1 immunotherapy.

The high heterogeneity of gastric cancer necessitates the identification of molecular subtypes to enable personalized immunotherapy. Several biomarkers associated with the tumor microenvironment (TME), including PD-L1 and microsatellite instability (MSI), have been explored to identify suitable candidates for immunotherapy^5^, ^6^. Nevertheless, their sensitivities and specificities differ and lack consistency; certain ones are even conflicting. Hence, up until now, there is no appropriate biomarker available for efficient patient categorization, and additional investigation is required to discover supplementary biomarkers or combinations of biomarkers that can better forecast clinical prognosis and reaction to immunotherapy.

The complex interaction between immunotherapy and immune cells that infiltrate tumors (TIICs) has become a fascinating and rapidly progressing field of study. Predicting tumor behavior has been suggested to be possible by examining the presence and dispersion of various immune cells^7^. Among the TIICs, memory T cells (MTCs), which constitute a distinct subgroup of CD4+ T helper (Th) cells, have garnered substantial attention owing to their potential contribution to effective anti-tumor immunity. Several research studies have found that memory T cells originating from tumor-responsive T cells display improved anti-tumor functionality in vitro and in vivo. This enhancement can possibly be attributed to their reduced activation thresholds, increased ability to migrate to lymph nodes, and extended persistence^8^. The prognosis of breast cancer, gastric cancer, lung adenocarcinoma, and pancreatic cancer is strongly linked to CD4+ memory T cells^9^. Several studies have utilized gene expression data and computational algorithms to analyze the immune cell composition in gastric cancer and found that gastric cancer subtypes enriched with CD4+ memory T cells had better prognosis^10^, ^11^. However, whether they can be used as predictors of the immunotherapy effect in gastric cancer remains unclear.

Our present study highlights the significance of activated CD4+ memory T cells, rather than the overall population of CD4+ memory T cells, in predicting the prognosis of patients with GC. We further established an activated CD4+ memory T cell-related risk score model based on BATF2, MYB, and CD36 expression. This model showed a better immune checkpoint blockage (ICB) response prediction ability than the CD8+PD-L1 model. The MYB\BATF2\CD36 model combined with CD8 and PD-L1 showed optimized prediction efficacy.

## Materials and methods

### Data sources

The transcriptomic information from the TCGA-STAD and GSE66229 datasets was acquired individually from The Cancer Genome Atlas (TCGA) and Gene Expression Omnibus (GEO) repositories. The TCGA-STAD dataset comprised 407 samples in total, comprising 375 tumor samples and 32 normal samples. A total of 337 gastric cancer (GC) samples were seen as a training set to build the prognostic model after excluding GC samples without survival data and those with a follow-up period of less than 30 days from the TCGA-STAD dataset. The external validation set consisted of microarray expression profiling data from 300 GC samples and 100 normal samples, known as the GSE66229 dataset.

Between 2012 and 2015, Zhongshan Hospital gathered Formalin-fixed, paraffin-embedded (FFPE) blocks from 80 STAD patients who underwent surgical resection prior to chemotherapy or radiation therapy. On June 12, 2021, the most recent follow-up took place, with a median follow-up duration of 1891 days (ranging from 527 to 3007 days). Between 2016 and 2022, Zhongshan Hospital obtained 100 more FFPE blocks from patients undergoing immunotherapy before starting ICB therapy. Every patient was diagnosed with advanced stomach cancer using gastroscopy and underwent at least one round of immunotherapy until the disease progressed or unacceptable toxicity appeared. The mRECIST criteria^12^. This study was approved by the Zhongshan Hospital Research Ethics Committee (B2022-537), and all patients provided written informed consent.

### WGCNA and DEG analysis

To identify genes related to immune cells (IRG), the Weighted Gene Co-expression Network Analysis (WGCNA) R package was utilized 13, 14. To guarantee a network that follows a power-law distribution, a gentle threshold was established. Co-expression matrices were used to group genes into modules, and correlation analysis was employed to screen the key modules that were related to immune cells most closely. In the two datasets, the comparison of differential gene expression (DEG) between GC and normal samples was conducted using the DESeq2 R package^14^.

### Characteristic of high/low immune-score groups

To further observe the clinical and biological characteristic in each group, an analysis of Gene Ontology (GO) and Kyoto Encyclopedia of Genes and Genomes (KEGG) enrichment using clusterProfiler R packages was conducted to identify biological mechanisms; the MCPcounter R package was used to calculate the degree of immune microenvironment infiltration; the online tool TIDE was applied to determine the TIDE score and assessing the differences in immunotherapy sensitivity.

### Other materials and methods

Details of analysis of immune cell infiltration landscape, constructing and validating of immune-score prognostic model, the cell lines, T cell activation assay, RNA transfection, quantitative polymerase chain reaction (qPCR), western blot, immunohistochemistry, multiplexed immunofluorescence, co-immunoprecipitation (co-IP), enzyme-linked immunosorbent assay (ELISA) and cell proliferation assay are described in the **Supplementary materials and methods**.

### Statistical analysis

The categorical variables were presented as frequencies and percentages, while continuous variables were described using means with standard deviations (SDs) or medians with interquartile ranges (IQRs). To compare the means of continuous variables that were normally distributed, independent group t-tests were utilized. For data that did not follow a normal distribution, the Mann-Whitney U test was employed instead. One-way analysis of variance (ANOVA) was employed to assess differences among multiple groups, and for independent data, post hoc Dunnett's multiple comparison test or Tukey's multiple comparisons was conducted. When comparing more than two groups and variables, a two-way ANOVA was conducted, followed by Sidak's multiple comparisons test. The chi-square or Fisher's exact tests were used to compare categorical variables. The Spearman correlation analysis was utilized to conduct correlation analyses. The Kaplan-Meier method was utilized to draw survival curves, and the log-rank test was employed to compare survival distributions. A *p*-value less than 0.05 was used to determine statistical significance. R, version 4.1.0 was utilized for performing data analysis and visualization.

## Results

### Activated CD4+ memory T cells associated with favorable prognosis of GC patients

**Figure 1:**
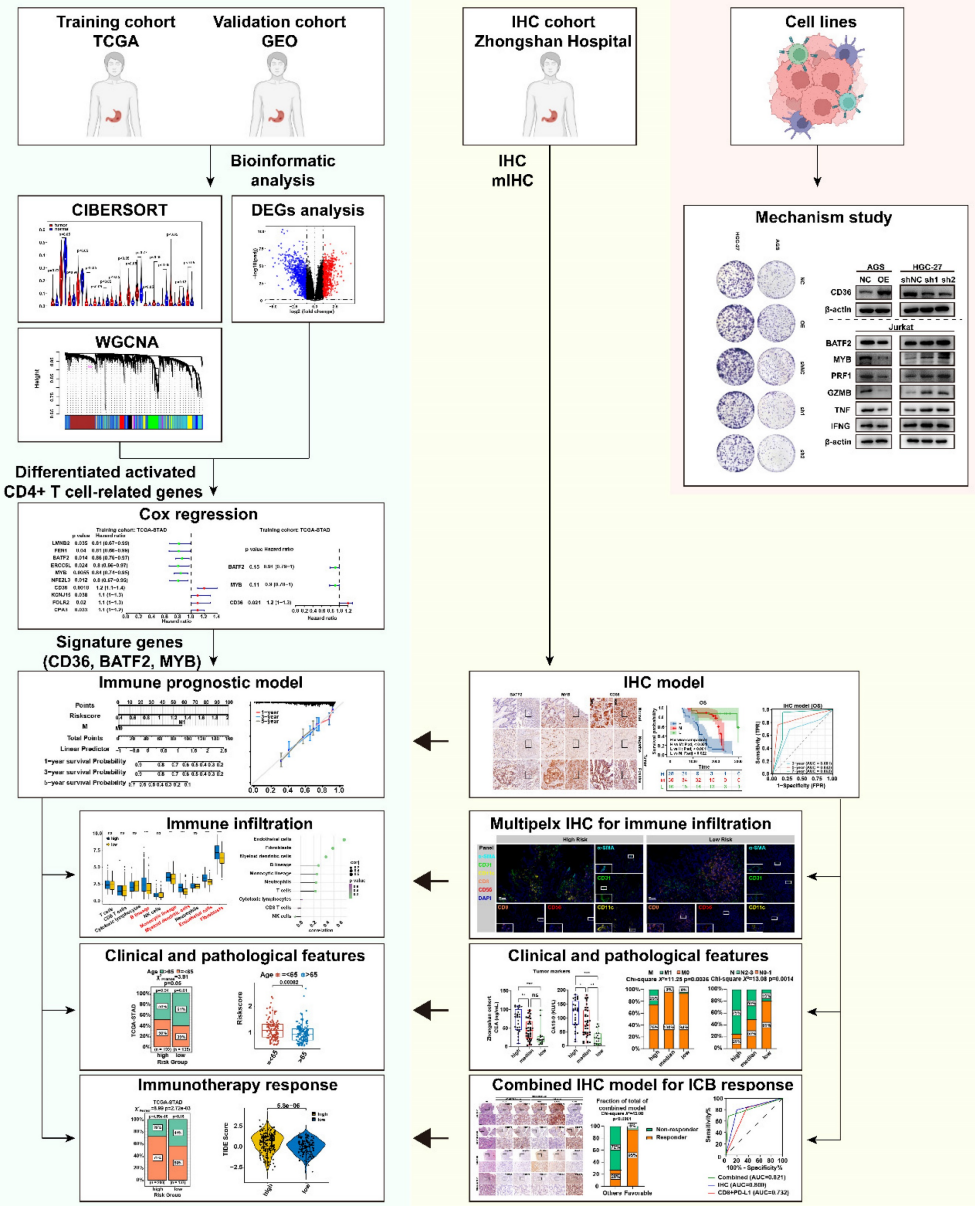
** Flowchart of the study.** This study utilized the TCGA gastric cancer dataset as the development set and the GEO gastric cancer dataset GSE66229 as the validation set. The CIBERSORT algorithm was used to calculate the proportion of immune cell infiltration, and immune cell-related genes were screened through weighted gene co-expression network analysis (WGCNA). Univariate Cox and Stepwise regression analyses were used to identify prognostic genes and construct a prognostic model, which was further explored in relation to immune cell infiltration, clinicopathological features, and immunotherapy sensitivity. The effectiveness of the model in predicting the prognosis and immune therapy response was verified using immunohistochemical (IHC) and multiplex immunofluorescence (mIHC) staining in a gastric cancer patient cohort from Zhongshan Hospital, and corresponding molecular mechanisms were explored through cellular experiments.

**Figure 2:**
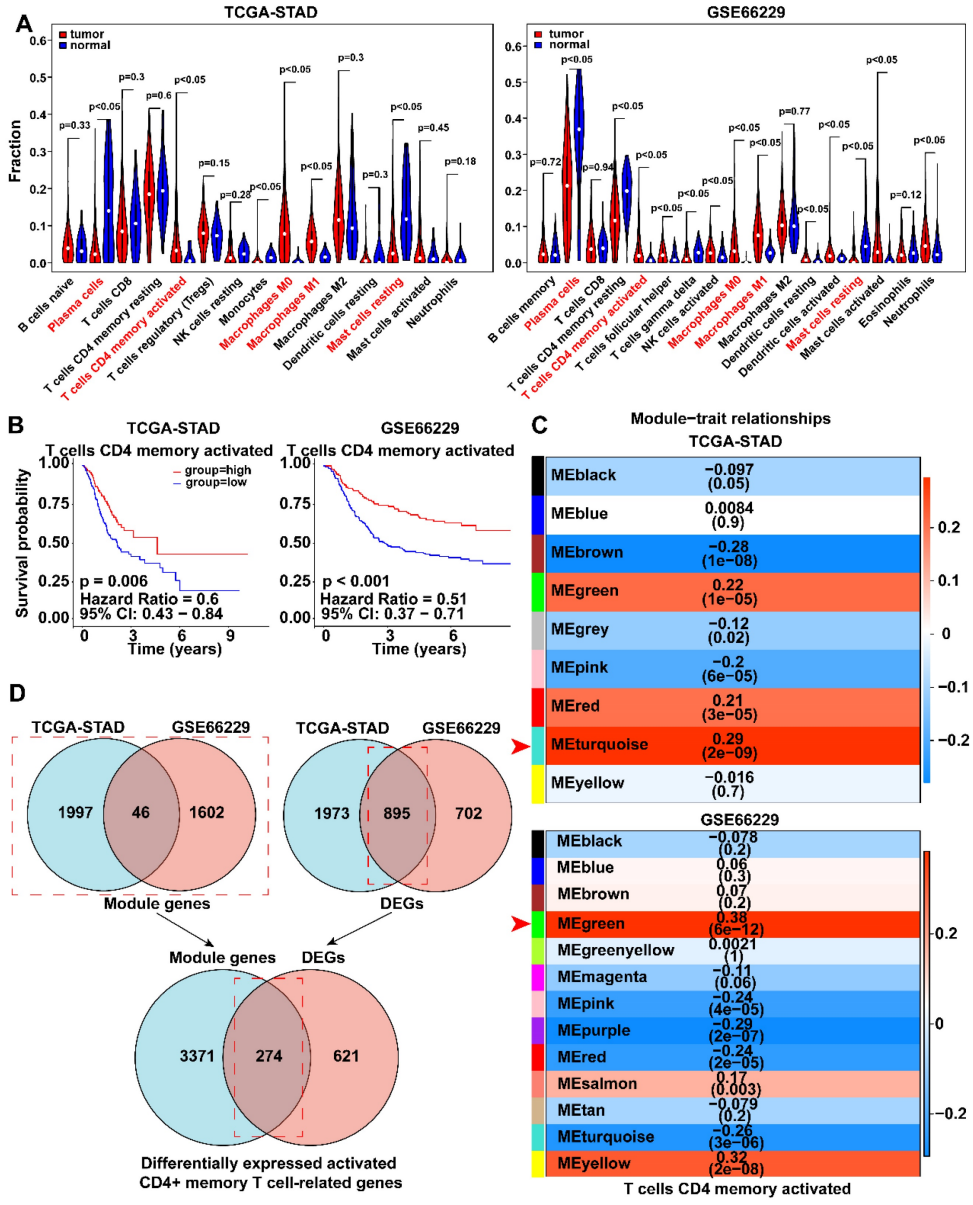
** Analysis of immune cells infiltration and the Kaplan-Meier curve for survival based on immune cells. A** Violin plots showed the differentially expressed immune cells in tumor and normal groups of TCGA-STAD and GSE66229 datasets. Red fonts indicated the immune cells with significant infiltration difference in each dataset. **B** Kaplan-Meier survival curves showed the survival rate of patients with highly expressed activated CD4+ memory T cells was higher than that of those with lowly expressed activated CD4+ memory T cells. Survival distributions were compared using the log-rank test. **C** Module-trait relationship between clustered modules and activated CD4+ memory T cells. Each row corresponds to a module Eigen gene, and the column to the status of activated memory CD4+ T cells. The numbers in each cell represent the corresponding correlation and p-value. **D** Venn diagram showed 274 differentially expressed genes associated with activated CD4+ memory T cells were found.

The workflow chart was presented in **Figure 1**. The CIBERSORT algorithm was employed to calculate the proportion of each immune cell in TCGA-STAD and GSE66229 datasets after excluding immune cells with zero percentage in more than half of the samples **(Fig. S1A)**. We found that activated memory CD4+ T cells, M0 macrophages, and M1 macrophages were significantly enriched, whereas plasma cells and resting mast cells were significantly decreased in GC (*p* < 0.05) **(Fig. 2A)**. K-M analysis further revealed that patients with more abundance activated CD4+ memory T cells had a significantly better prognosis (HR 0.51, 95% CI 0.37-0.71, *p* < 0.001) **(Fig. 2B, Fig. S1B, C)**.

### Establishment of an activated CD4+ memory T cell-related risk score model based on BATF2, MYB and CD36

**Figure 3:**
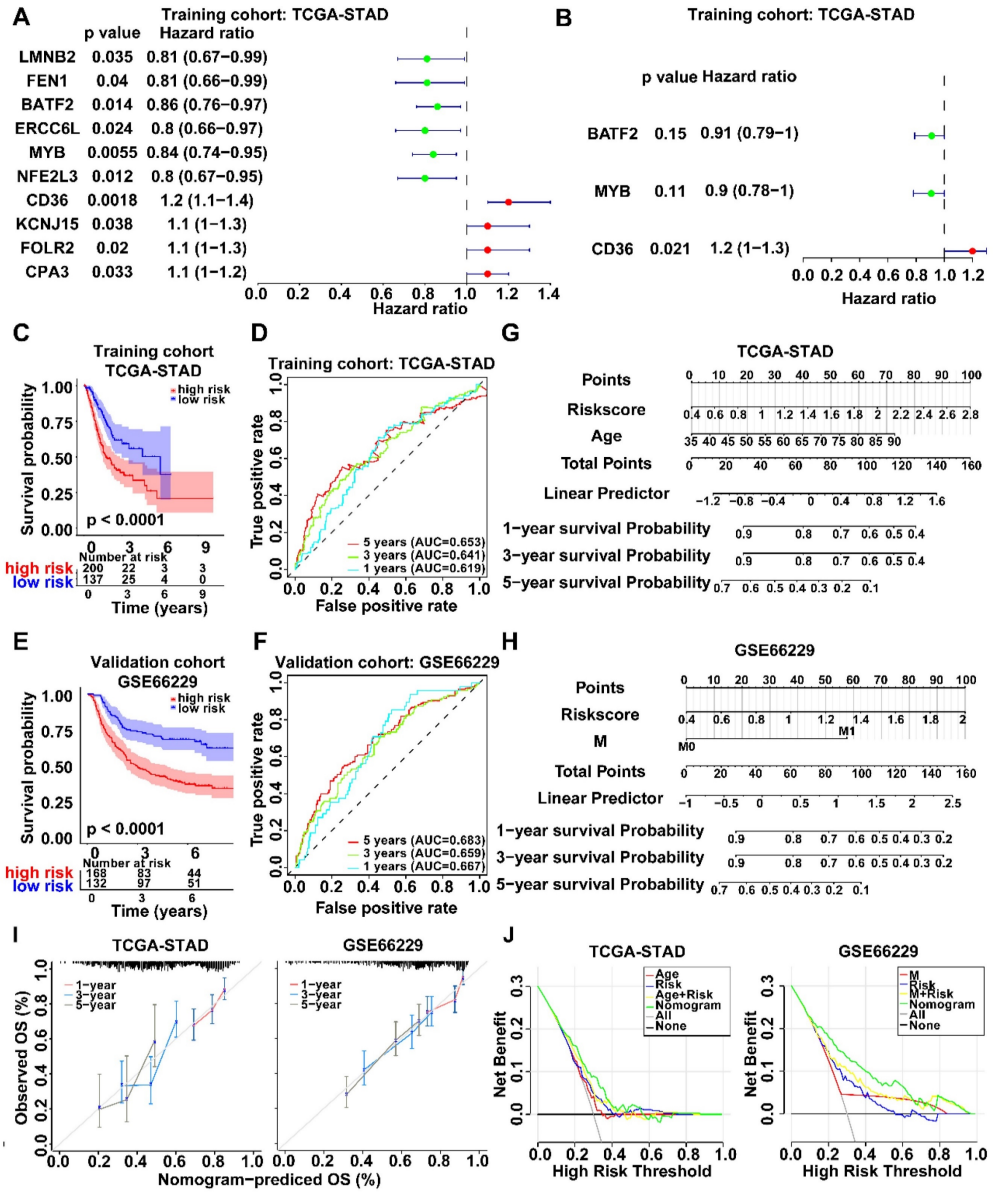
** Identification of survival-related genes and establishment of a new risk score prognosis system. A, B** Forest plots of survival-related genes by univariate and multivariate Cox regression. 3 key prognosis genes were screened out using Stepwise regression and an immune cell-related model for prognosis was established. **C, E** Kaplan-Meier survival curves of both groups with high and low risk score in TCGA-STAD and GSE66229 datasets. Survival distributions were compared using the log-rank test. **D, F** ROC curves of prediction model based on 3 key prognosis genes showed the favorable accuracy and specificity. **G, H** Nomogram to estimate the survival probability of GC patients in both datasets. **I** Calibration curves revealed the predictive accuracy of nomogram. **J** DCA curves comparing nomogram and clinical parameters. The y-axis represented net benefits and the x-axis measured threshold probability.

WGCNA was employed to identify gene modules of correlated genes highly associated with activated CD4+ memory T cell. After determining the optimal soft threshold **(Fig. S2)**, 9 and 13 gene modules for TCGA-STAD and GSE66229 datasets were identified. Among them, the turquoise module (TCGA cohort) and the green module (GSE66229 cohort) had the highest correlation with activated CD4+ memory T cells (r = 0.29, *p* <0.001; r = 0.38, *p* <0.001, respectively) **(Fig. 2C).** Finally, 3645 related module genes were selected**,** and DEG analysis was performed to select 895 DEGs from the two datasets **(Fig. 2D)** based on |log_2_FC| > 1 and an adjusted *p* value < 0.05. Ultimately, 274 genes associated with activated CD4+ memory T cells were obtained **(Fig. 2D)**. Through univariate and multivariate Cox regression analyses of a panel of 274 genes, we identified a robust 3-gene risk score model consisting of BATF2, MYB, and CD36 **(Fig. 3A, B)**. The immune risk score = (-0.097) * BATF2 + (-0.11) * MYB + 0.16 * CD36. Univariate and multivariate COX analyses showed that the risk model had an independent prognostic value **(Fig. S3)**.

According to the TCGA cohort, patients with higher scores had significantly worse overall survival than those with lower scores (*p* < 0.001) **(Fig. 3C)**. The prognostic model demonstrated favorable performance with an area under the ROC curves (AUC) of 0.619, 0.641, and 0.653 at 1, 3, and 5 years, respectively **(Fig. 3D)**. We also observed similar results in the validation GSE66229 cohort **(Fig. 3 E, F)**. After univariate and multivariate COX regression, a nomogram integrating the risk score and other independent clinicopathological variables was further constructed to accurately predict GC prognosis **(Fig. 3 G, H)**. Calibration curves and DCA showed the best clinical practicality for prognosis prediction in patients with GC. The validation cohort GSE66229 also showed the same results **(Fig. 3 I, J)**.

**Figure 4:**
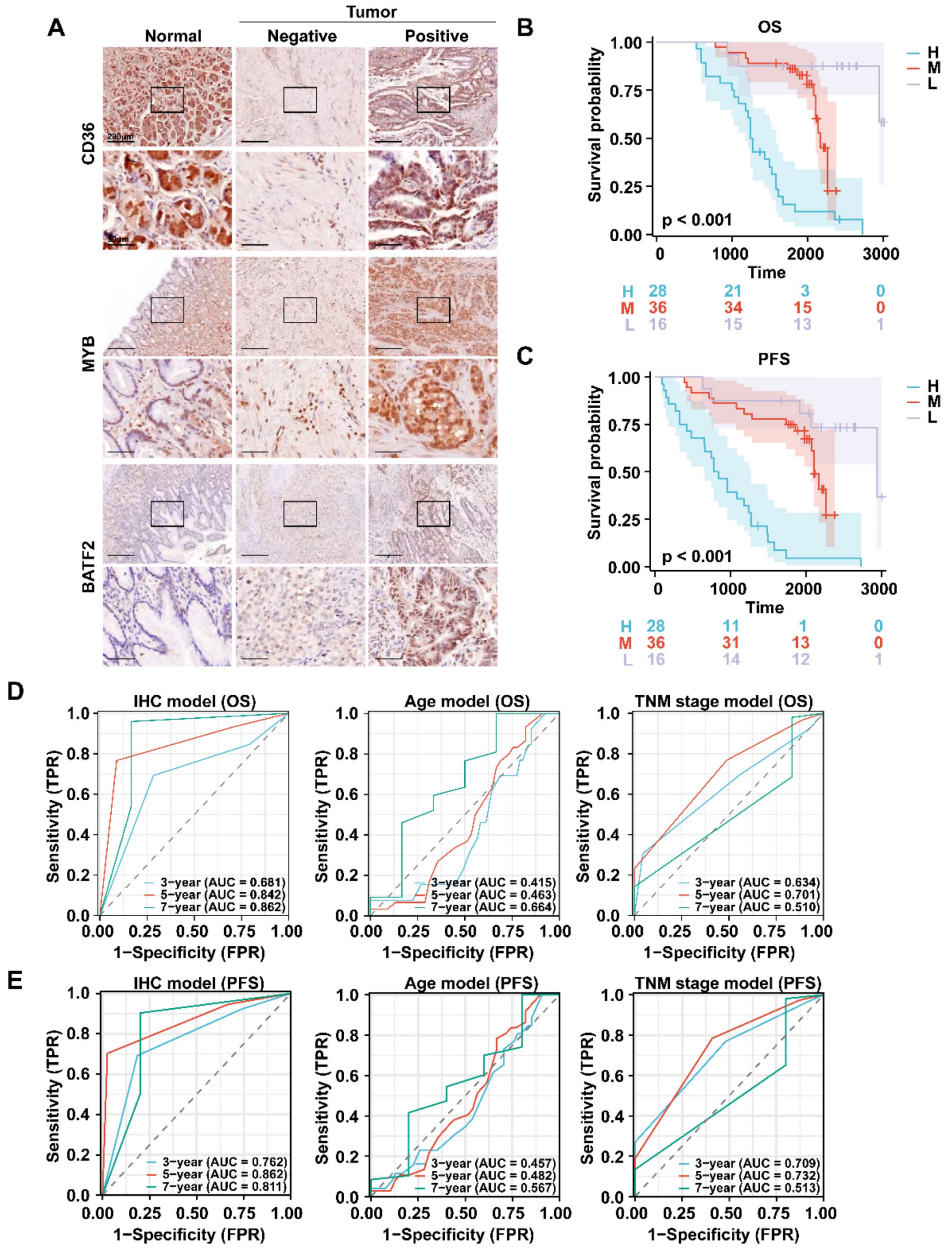
** Validation of the efficacy of a prognostic model in clinical specimens. A** Immunohistochemistry staining of CD36, MYB and BATF2 in GC and paired para-cancerous tissues (n = 80). Scale bar equals to 200μm (high power lens) and 50μm (low power lens) **B, C** Kaplan-Meier analysis for OS and RFS in GC patients with different risk score. Survival distributions were compared using the log-rank test. “H”, “M” and “L” stands for “High risk group”, “Median risk group” and “Low risk group”. **D, E** ROC curves of the IHC model, age model, and TNM stage model in predicting OS, RFS.

We also collected 80 pairs of gastric cancer and corresponding para-cancerous tissue samples from Zhongshan Hospital for immunohistochemical staining as Zhongshan cohort. Since CD36 was highly expressed in the high-risk group, and BATF2 and MYB were lowly expressed in the high-risk group, we defined CD36+ MYB- BATF2- patients as the high-risk group (n = 28), CD36- MYB+ BATF2+ patients as the low-risk group (n = 16), and other patients as the medium-risk group (n = 36) **(Fig. 4A)**. We found that the high-risk group had the lowest overall or progression-free survival (PFS) rates **(Fig. 4B, C)**. Compared with the mRNA level model, the ROC curves showed that the IHC model had better 3-year and 5-year AUC for OS (0.681 vs. 0.641 vs. 0.659; 0.842 vs. 0.653 vs 0683). It also exhibited superior prognosis prediction ability than age and TNM stage models in predicting OS and PFS at 3 years (OS:0.681 vs. 0.415 vs. 0.634; PFS:0.781 vs. 0.453 vs. 0.724), 5 years (OS:0.842 vs. 0.463 vs. 0.701; PFS:0.870 vs. 0.485 vs. 0.739), and 7 years (OS:0.862 vs. 0.664 vs. 0.510; PFS:0.836 vs. 0.593 vs. 0.515) **(Fig. 4D, E)**.

### CD36+ MYB- BATF2- signature based high-risk group suffered more aggressive GCs and had a more suppressive tumor microenvironment

The heatmaps of the correlation between each clinical characteristic and the risk score of the two datasets were shown in **Fig. S4A, B**. Based on the clinical information provided by TCGA-STAD and GSE66229, the stage, age, T, and N were significantly correlated with the risk score (**Fig. S4C-F, Table S1, 2,** *p* < 0.05), showing that patients with higher risk tend to be younger and have a more severe and invasive type of gastric cancer. Based on the 1339 DEGs between the two group samples in the two datasets **(Fig. S5A)**, GO and KEGG analysis was performed. Top10 GO terms for each category are shown in **Fig. S5B**. Meanwhile, there are 25 KEGG pathways that were enriched**,** including some linked to gastric cancer, like 'Wnt signaling pathway' and 'focal adhesion', as shown in **Fig. S5C**.

To investigate the differences in the tumor immune microenvironment (TME) between low- and high-risk GC samples, the scores of eight immune cells and two stromal cells of GC samples were calculated in two datasets. The results showed that five cell subtypes (monocytic lineage, B lineage, endothelial cell myeloid dendritic cells, and fibroblasts) were enriched in the high-risk group of the TCGA-STAD dataset significantly and seven cell subtypes (T cells, monocytic lineage, B lineage, myeloid dendritic cells, Endothelial cells, Neutrophils, and fibroblasts) were enriched in the high-risk group of the GSE66229 dataset significantly** (Fig. S5D,** *p* < 0.05**)**. Among the above immune cells, myeloid dendritic cells, endothelial cells, and fibroblasts were positively correlated with the risk score (cor > 0.3 and *p* < 0.05), whereas CD8+ T cells and natural killer cells showed a mild negative correlation with the risk score** (Fig. S5E)** in both datasets, indicating the formation of a suppressive TME unfavorable for immunotherapy^15^ in BATF2, MYB and CD36 based high-risk GC patients.

**Figure 5:**
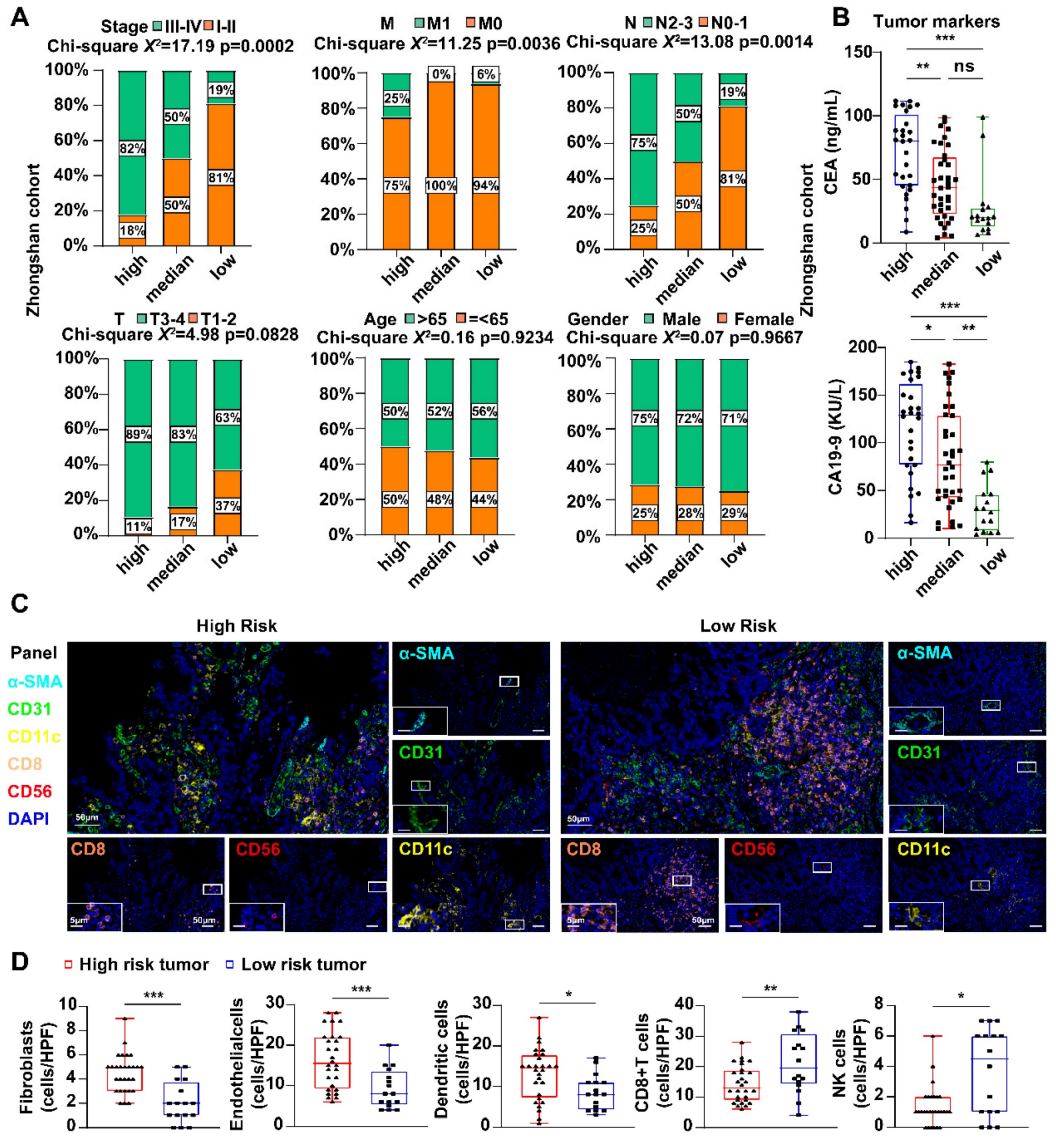
** Differences in clinical features and tumor microenvironment in the IHC cohort. A** The percentage of different clinical features among different risk score groups. Statistical analyses: Chi-square test and Fisher's exact test. **B** Scatter plots showed the expression of CEA and CA19-9 among different risk score groups. Statistical analyses: One-way ANOVA and Tukey's multiple comparisons test. **C** mIHC images showed the staining for fibroblast (α-SMA, cyan), endothelial cells (CD31, green), dendritic cell (CD11c, yellow), CD8+ T cell (CD8, orange) and NK cell (CD56, red) in GC tissues between high and low risk groups. Scale bar equals to 50μm and 5μm. **D** Scatter plots showed the density of fibroblast, endothelial cells, dendritic cell, CD8+ T cell and NK cell among different risk score groups. Statistical analyses: Unpaired two-tailed Student's t-test and or Mann Whitney test. ns indicated no statistically significance, *p<0.05, **p<0.01, *** p<0.001.

In the Zhongshan cohort, clinical information showed that stage and TNM (N and M) varied among the different risk groups **(Fig. 5A)**, which verified the results based on mRNA level. The high-risk group of patients showed higher expression of tumor markers related to gastric cancer, including carcinoembryonic antigen (CEA) and carbohydrate antigen 199 (CA199) **(Fig. 5B)**. Immune cell enrichment or decrease in high-risk GC patients was validated using multiplex immunofluorescence. As depicted in **Fig. 5C, D**, the expression levels of SMA (a marker for fibroblasts), CD31 (a marker for endothelial cells), and CD11c (a marker for myeloid dendritic cells) were significantly elevated in the high-risk group than the low-risk group. These findings align with those results obtained from the analysis of high- and low-risk groups in publicly available databases. CD8+T cells (CD8) and natural killer cells (CD56) were relatively impoverished in high-risk samples.

### MYB\BATF2\CD36 model combined with CD8 and PD-L1 for selecting patients suitable for immunotherapy

**Figure 6:**
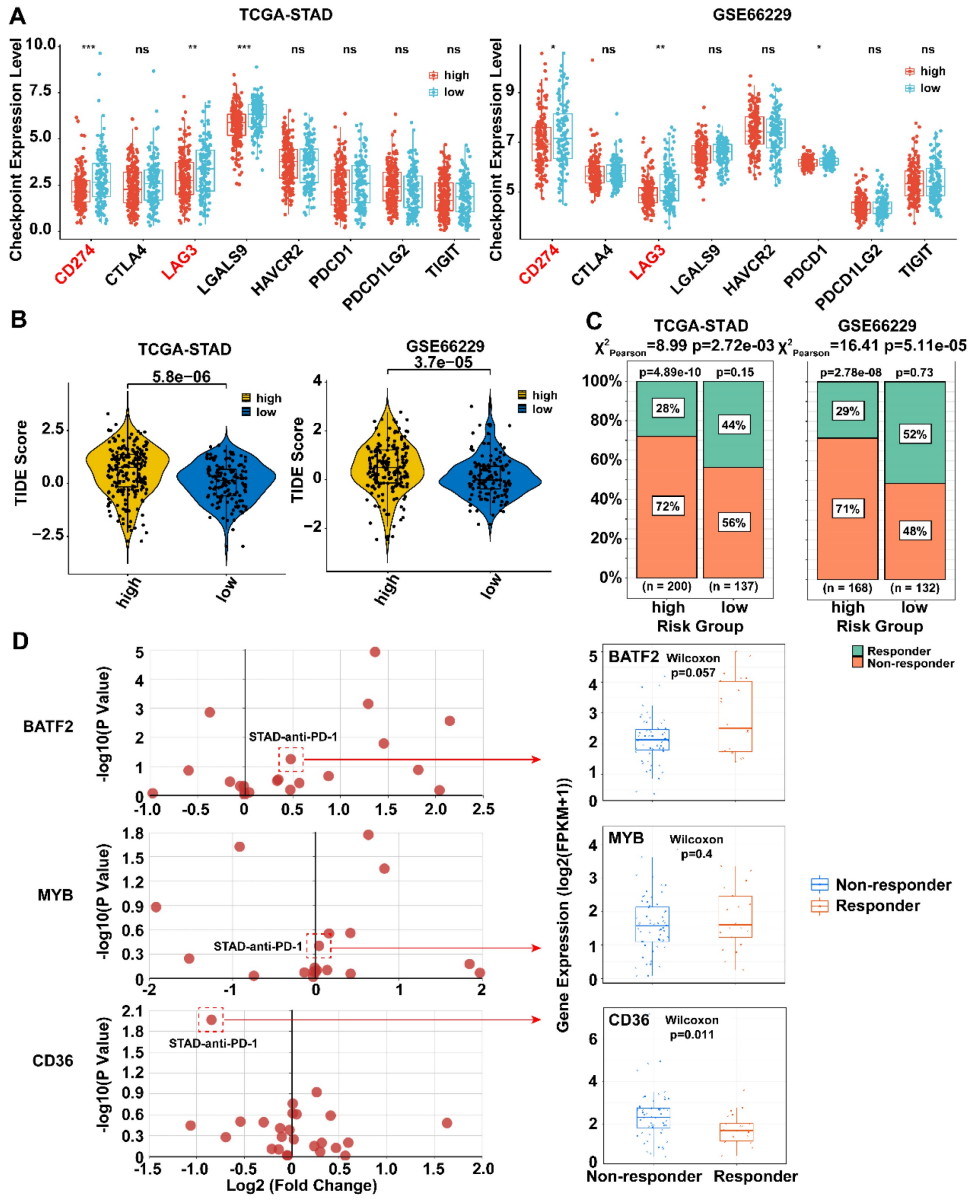
** Therapeutic benefits of risk scores calculated by prognosis model. A** Boxplots showed the immune checkpoints related genes expression in high- and low- risk score groups. Statistical analyses: Mann Whitney test. **B** Violin plot illustrated the TIDE scores for patients with gastric cancer across risk score groups. Statistical analyses: Mann Whitney test. **C** Percentage bar charts showed the percentage of immunotherapy responders and non-responders between high- and low-risk group. Statistical analyses: Chi-square test. **D** The association between three model genes (CD36, BATF2 and MYB) and immunotherapy response. Boxplots showed the expression levels of three model genes between anti-PD-1 responders and non-responder. Statistical analyses: Wilcoxon test. ns indicated no statistically significance, *p<0.05, **p<0.01, *** p<0.001.

Next, we compared the expression of eight immune checkpoints (PD-L1 (CD274), PD-L2 (PDCD1LG2), LAG-3 (LAG3), CTLA-4 (CTLA4), GAL9 (LGALS9), TIM-3 (HAVCR2), PD-1 (PDCD1), and TIGIT) between the high- and low-risk groups in the TCGA-STAD and GSE66229 datasets. The results showed that PD-L1 and LAG-3 were highly expressed in the low-risk group in both datasets (*p* < 0.05) **(Fig. 6A).** Low-risk group had lower TIDE scores and more ICB responders in the two datasets **(Fig. 6B, C,** *p* < 0.05**)**, which further implying that low-risk patients may be more sensitive to immunotherapy.

We next examined the association between the three model genes (CD36, BATF2 and MYB) and immunotherapy response using datasets composed of pre-treated tumor biopsies from responders and non-responders in the TIGER database. MYB and BATF2 were highly expressed in responders, whereas CD36 showed opposite results in most datasets that contained a STAD anti-PD-1 treatment dataset^4^ **(Fig. 6D)**.

**Figure 7:**
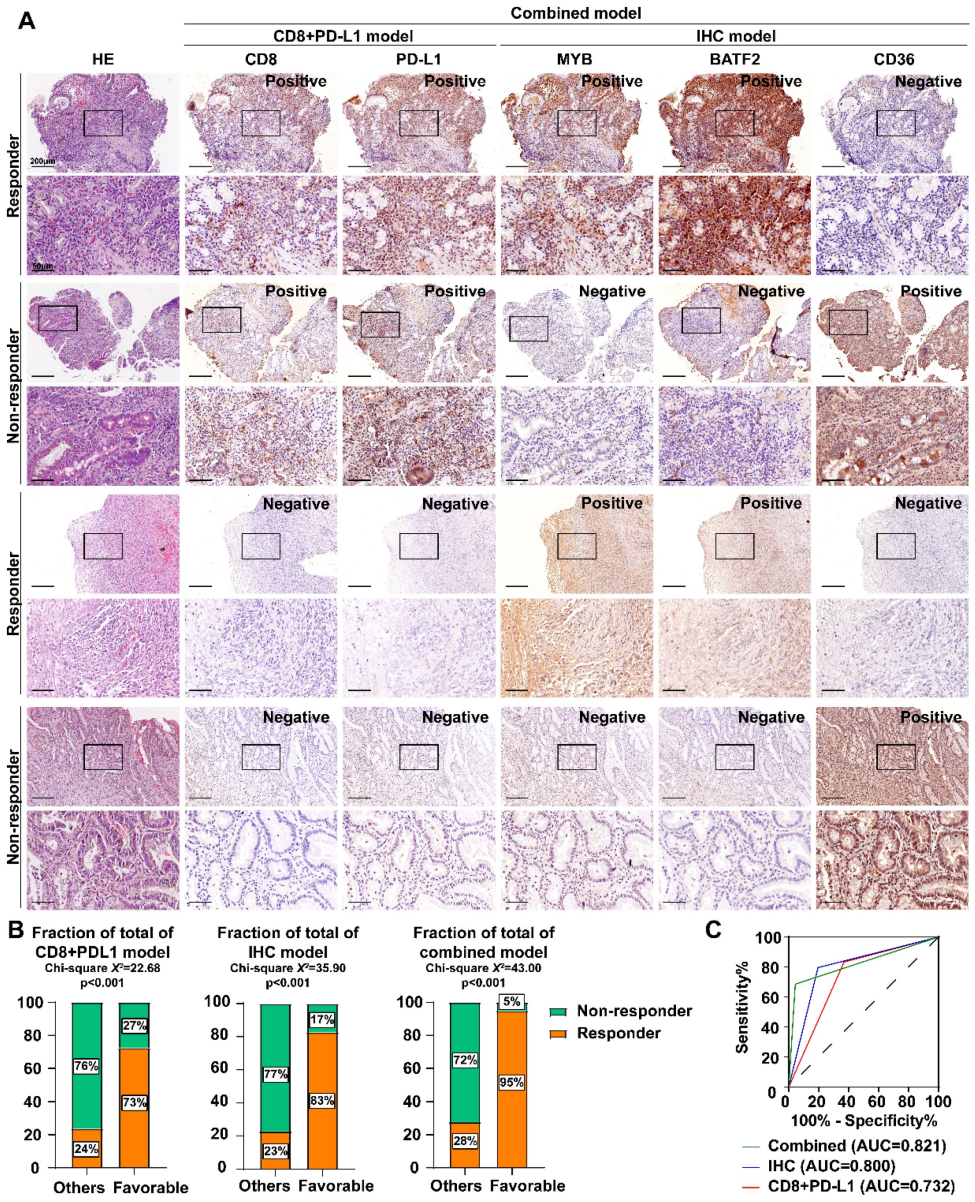
** Establishment of BATF2/MYB-related IHC scoring system for sensitivity to immunotherapy. A** Immunohistochemistry and HE staining of CD8, PD-L1, MYB, BATF2 and CD36 in gastroscope samples from GC patients (n = 100). Scale bar equals to 200μm (high power lens) and 50μm (low power lens) **B** The percentages of responders and non-responders between different model groups. Statistical analyses: Chi-square test. **C** ROC curves of three different ICB prediction IHC model.

Therefore, we established an MYB\BATF2\CD36-related-IHC model and evaluated its specificity and sensitivity in predicting ICB efficacy compared to traditional PD-L1 or CD8 IHC. One hundred patients receiving ICB after the diagnosis of advanced gastric cancer (54 responders and 46 non-responders) were separated according to the IHC staining score of CD8, PD-L1, MYB, BATF2 and CD36: CD8+PDL1 model favorable group (CD8+ PD-L1+, n = 62) and others (n = 38); IHC model favorable group (MYB+ BATF2+ CD36-, n = 52), and others (n = 48); combined model favorable group (CD8+ PD-L1+ MYB+ BATF2+ CD36-, n = 39) and others (n = 61). We found that some patients who were traditionally defined as ICB-favorable (CD8+ PD-L1+) expressed low MYB, low BATF2, or high CD36 were finally proven to be non-responders. Those defined as unfavorable (CD8- PD-L1-) may respond well to ICB if they express high MYB, high BATF2, and low CD36 **(Fig. 7A)**. Our IHC model (Chi-square *X^2^*=35.90, *p* < 0.001; AUC = 0.800) showed better ICB response prediction ability than the CD8+ PD-L1 model (Chi-square *X^2^*=22.68, *p* < 0.001; AUC = 0.732). The combined model (MYB\BATF2\CD36 model combined with CD8 and PD-L1, Chi-square *X^2^*=43.00, *p* < 0.001; AUC=0.821) showed optimized prediction efficacy (68.52% sensitivity and 95.65% specificity) **(Fig. 7B, C)**.

### CD36 repressed the activation of CD4+ memory T cells via inhibiting CXCL3-BATF2\MYB axis

As our model was based on activated CD4+ memory cells, we further investigated the association between the three model signatures and activated CD4+ memory T cells.

**Figure 8:**
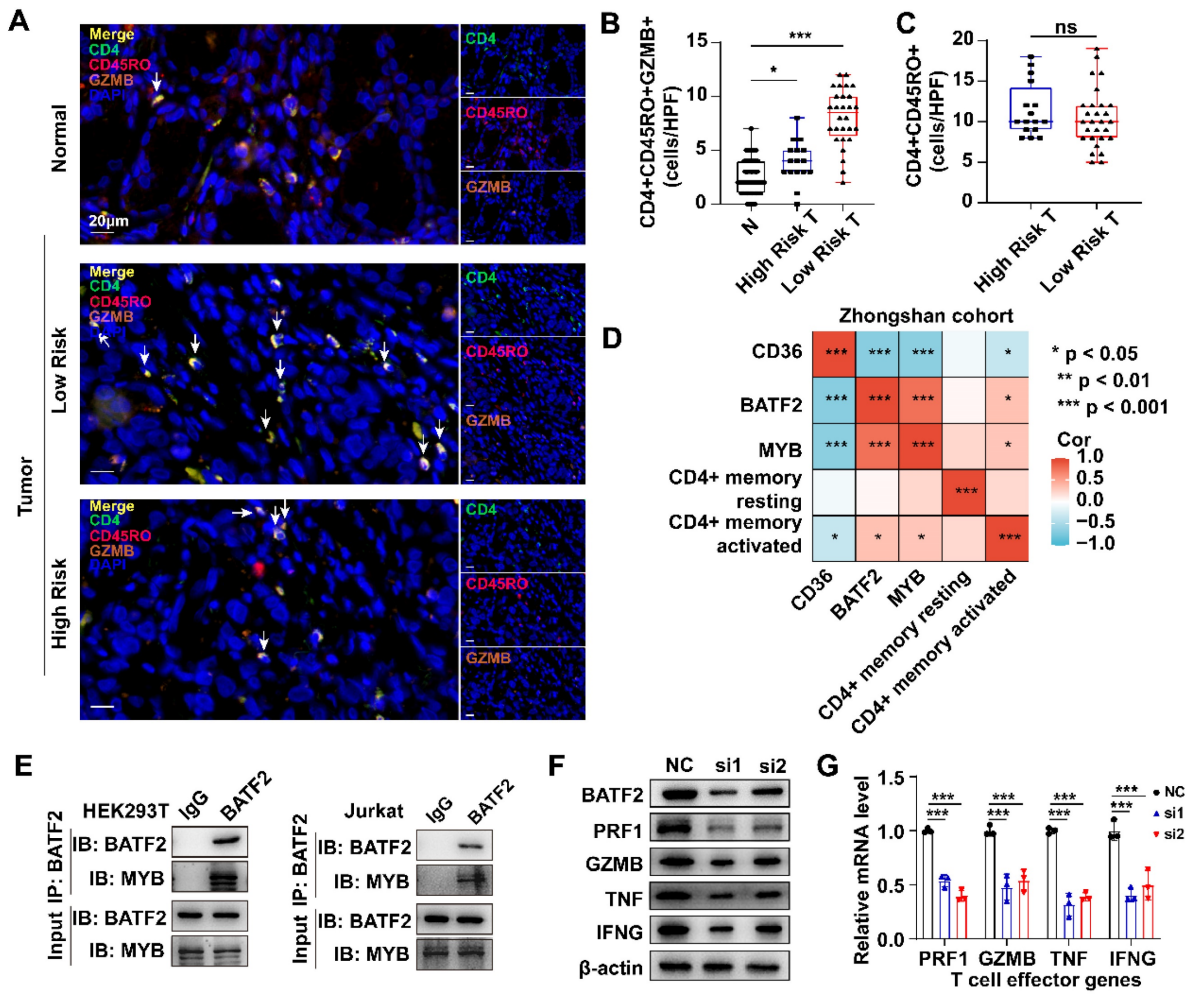
** Three model genes correlated with the activation of CD4+ memory T cells. A-C** mIHC images and boxplots revealed the high-risk samples had fewer CD4+ memory activated T cells (CD4+ CD45RO+ GZMB+) than the low-risk sample and no significant difference in total CD4+ memory T cells (CD4+ CD45RO+). Scale bar equals to 20μm. Statistical analyses: One-way ANOVA and Dunnett's multiple comparisons test and Unpaired two-tailed Student's t-test. **D** Correlation between IHC score of CD36, BATF2 and MYB and infiltration of resting or activated CD4+ memory T cells using Spearman analysis in 44 Zhongshan cohort samples.** E** CO-IP assay in HEK293T and Jurkat T cells detected exogenous and endogenous interaction between BATF2 and MYB. Each experiment was independently repeated three times. **F** Western blot analysis of PRF1, GZMB, TNF, IFNG expression in cell line Jurkat T cells after knocked out of BATF2. Each experiment was independently repeated three times. **G** qPCR analysis of PRF1, GZMB, TNF, IFNG relative mRNA expression in Jurkat T cells after knocked out of BATF2, n = 3. Statistical analyses: Two-way ANOVA and Dunnett's multiple comparisons test. ns indicated no statistically significance, *p<0.05, **p<0.01, *** p<0.001.

Using the TIMER2.0 database, we found that MYB and BATF2 were positively correlated with the abundance of activated CD4+ memory T cells but had no correlation with the abundance of resting CD4+ memory T cells or naïve CD4+ T cells, whereas CD36 showed little impact on either type of CD4+ T cells **(Fig. S6A)**. Using the TIGER database, we found that CD36 had a positive correlation with T cell dysfunction (R = 0.43, *p* < 0.001), BATF2 was positively correlated with T cell-inflamed GEP (R = 0.58, *p* < 0.001) and negatively correlated with T cell exclusion (R = -0.23, *p* < 0.001), and MYB was negatively correlated with T cell dysfunction (R = -0.22, *p* < 0.001) and exclusion (R = -0.22, *p* < 0.001) **(Fig. S6B)**. Next, we calculated the correlation between BATF2, MYB and classical T cell effector molecules in the TCGA-STAD dataset. As shown in **Fig. S6C**, BATF2 and MYB were significantly correlated with IFNG, GZMB, PRF1 and TNF **(Table S3)**. These results indicated that CD36, BATF2 and MYB might influence the activation of CD4+ memory T cells via a classical T cell activation pathway. Consistent with the above results, we found that high-risk samples had fewer activated CD4+ memory T cells than low-risk samples by multiplex immunofluorescence staining (CD4\CD45RO\GZMB)^16^ **(Fig. 8A, B)**. However, there was no significant difference in the total CD4+ memory T cells (CD4+\CD45RO+) between the two groups **(Fig. 8C)**. Spearman correlation analysis using the quantitative data above in Zhongshan cohort also confirmed the positive correlation between activated CD4+ memory T cells and MYB and BATF2 (Cor = 0.31 and 0.33, respectively) and the negative correlation between activated CD4+ memory T cells and CD36 (Cor = -0.35). Meanwhile, all the three genes had no significance correlation with resting CD4+ memory T cells **(Fig. 8D)**.

MYB is predominantly found in immune cells, and BATF2 is expressed both in malignant cells and immune cells according to the same SC-Seq dataset above **(Fig. S7A)**. BATF2 is predicted to enhance DNA-binding transcription factor activity. We hypothesized that BATF2 might interact with MYB to enhance its transcriptional activity. The interaction was confirmed using the STRING database **(Fig. S7B)** and CO-IP experiments in HEK293T cells and Jurkat T cells **(Fig. 8E)**. As an important transcription factor, MYB was predicted to bind to promoters of IFNG, GZMB, PRF1 and TNF in a ChIP-Seq dataset (GSM1442006) obtained from the Jurkat T cell line^17^ using the Cistrome Data Browser^18^, ^19^ **(Fig. S7C, D)**. The decrease in BATF2 by small interfering RNA (siRNA), represented by “si1” and “si2” in Figure, in T cells was shown to reduce the expression of effector molecules at both protein level and mRNA level **(Fig. 8F, G)**. These results confirm our hypothesis that BATF2 might activate the transcriptional activity of MYB to promote T cell activation.

**Figure 9:**
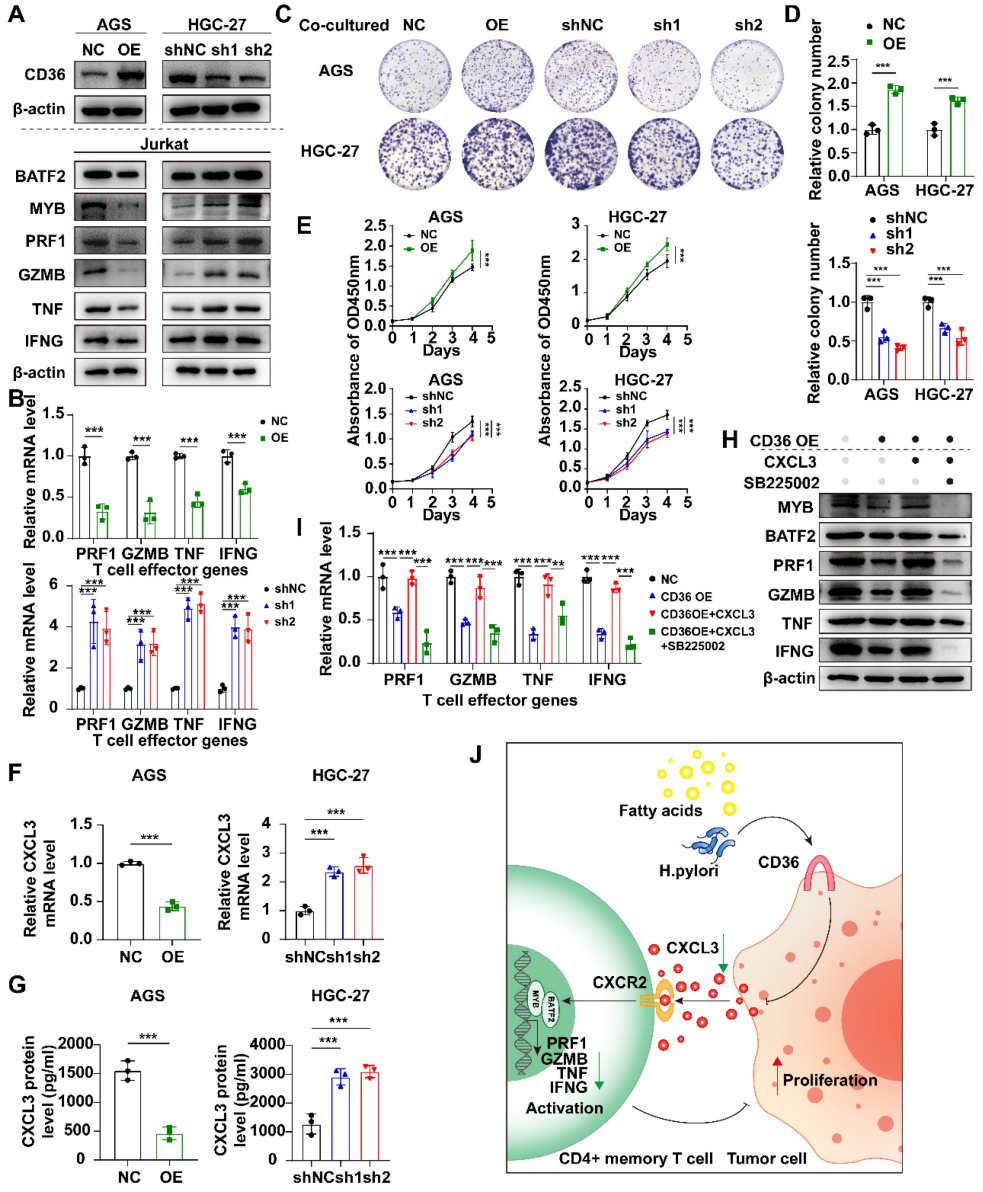
** Overexpression of CD36 inhibited CXCL3 secretion of gastric cancer cells and impaired T cell function. A** Western blot analysis of BATF2, MYB and T cell effector molecules (PRF1, GZMB, TNF, IFNG) in Jurkat T cells co-cultured with CD36 overexpressed or knock-downed gastric cancer cells. The experiment was repeated three times. **B** qPCR analysis of BATF2, MYB and T cell effector genes (PRF1, GZMB, TNF, IFNG) in Jurkat T cells co-cultured with CD36 overexpressed or knock-downed gastric cancer cells, n=3. Statistical analyses: Two-way ANOVA and Dunnett's multiple comparisons test, Two-way ANOVA and Šídák's multiple comparison test. **C, D** After co-culturing with T cells, gastric cancer cells AGS and HGC-27 with CD36 overexpression and knockdown exhibited different abilities in colony formation. Relative colony numbers were counted and shown in H, n = 3. Statistical analyses: Two-way ANOVA and Dunnett's multiple comparisons test, Two-way ANOVA and Šídák's multiple comparison test. **E** CCK-8 assays showed after co-culturing with T cells, gastric cancer cells AGS and HGC-27 with CD36 overexpression and knockdown exhibited different abilities in proliferation. Statistical analyses: Two-way ANOVA and Dunnett's multiple comparisons test, Two-way ANOVA and Šídák's multiple comparison test. **F** Comparison of qPCR titers of CXCL3 mRNA in gastric cells AGS and HGC-27 with CD36 overexpression and knockdown, n=3. Statistical analyses: Unpaired two-tailed Student's t-test or One-way ANOVA and Dunnett's multiple comparisons test. **G** The CXCL3 protein levels in supernatant of gastric cancer cells, n=3. Statistical analyses: Unpaired two-tailed Student's t-test or One-way ANOVA and Dunnett's multiple comparisons test.** H** Western blot analysis of BATF2, MYB and T cell effector molecules (PRF1, GZMB, IFNG, and TNF) in Jurkat cells co-cultured with normal AGS, CD36 over-expressed AGS, CD36 over-expressed AGS adding CXCL3 (10 ng/ml) and CD36 over-expressed AGS adding CXCL3 (10 ng/ml) and SB225002 (12.5 μm) for 24 h. The experiment was repeated three times. **I** qPCR analysis of BATF2, MYB and T cell effector molecules (PRF1, GZMB, IFNG, and TNF) in groups of **H**, n = 3. Statistical analyses: Two-way ANOVA and Dunnett's multiple comparisons test, Two-way ANOVA and Šídák's multiple comparison test. **J** Proposed schematic of CD36-CXCL3-BATF2\MYB signaling pathway regulatory mechanisms in activating T cells. Under the stimulation of a high-fat diet and Helicobacter pylori infection, CD36 receptors on gastric cancer cells were upregulated. This led to a reduction in the secretion of CXCL3. As a consequence, the transcription of T cell effector molecules PRF1, GZMB, IFNG, and TNF by the BATF2-MYB complex was affected, resulting in the suppression of CD4+ memory T cell activation. This finally impaired its suppression on proliferation ability of tumor cells. *p<0.05, **p<0.01, *** p<0.001.

Unlike MYB and BATF2, CD36 was mainly expressed in malignant cells according to a gastric cancer SC-Seq dataset (GSE134520) using the TISCH2 database^20^ **(Fig. S8A)**. CD36 was highly expressed in gastric tissues in high-fat diet (HFD) group compared to the normal diet (ND) group in the GSE69306 dataset **(Fig. S8B)**. Infection with Helicobacter pylori could also increase the expression of CD36 when the stage reaches intestinal metaplasia (IM), both in gastric tissues and in malignant cells **(Fig. S8C, D)**. To study the effect of CD36, which is mainly located in malignant cells, we investigated the proliferation ability between CD36-overexpressed (OE), CD36-knockdowned (sh1 and sh2) and control group (NC and shNC respectively) in AGS and HGC-27 cells and found no significant difference **(Fig. S9)**. We next co-cultured Jurkat T cells with CD36 overexpressed or knockdowned gastric cancer cells. Co-cultured with CD36 overexpressed gastric cancer cells, Jurkat T cells down-regulated the expression of BATF2, MYB, and T cell effector molecules (PRF1, GZMB, TNF, and IFNG) **(Fig. 9A, B)** and enhanced the proliferation of gastric cancer cells **(Fig. 9C-E)**, indicating the indirect impact of malignant CD36 on gastric cancer development was caused by the down-regulation of T cell function.

CD36- TLR4/TLR6 are responsible for the secretion of various cytokines. The regulation of the assembly of a TLR4-TLR6 heterodimer, which activates the transcription of pro-inflammatory cytokines, was reportedly attributed to CD36^21^-^23^. We hypothesized that malignant CD36 might affect T-cell function through these cytokines. We discovered CXCL3, a cytokine negatively correlated with CD36-TLR4/TLR6, positively correlated two other signature genes (BATF2 and MYB), and positively correlated with four T cell activation markers (PRF1, GZMB, TNF, and IFNG) **(Fig. S8E)**, suggesting CD36 may impair its secretion and lead to the dysfunction of T cells. CXCL3 mRNA expression level decreased when CD36 was overexpressed **(Fig. 9F)**. CXCL3 protein levels in the supernatant of CD36 overexpressed gastric cancer cells also decreased **(Fig. 9G)**. The activation of T cells could be rescued by adding CXCL3 in the co-culture system only when its receptor CXCR2 was not blocked using SB225002, a CXCR2 specific blockage **(Fig. 9H, I)**. These results indicated the important role of the CD36-CXCL3-BATF2/MYB axis in activating T cells **(Fig. 9J)**.

## Discussion

Although anti-PD-1 therapy has been widely used for treating gastric cancer, an effective biomarker that could predict the therapeutic effect remains unclear. Here, we report that activated CD4+ memory T cells are elevated and associated with favorable outcomes in GC patients. Accordingly, we established an activated CD4+ memory T cell-related risk score model based on BATF2, MYB and CD36 expression. This model showed a better ICB response prediction ability than the traditional CD8+PD-L1 model. When combined with the CD8+ PD-L1 model, the combined model showed optimized prediction efficacy. Mechanistically, CD36 represses the activation of CD4+ memory T cells by impairing CXCL3-BATF2/MYB axis.

Immune cells, as crucial members of the TME, affect the progression of tumors and the efficacy of immunotherapy. Recent studies have established various IRGs signatures to predict prognosis and/or immunotherapy efficacy in GC^24^-^29^. These findings indicated that the correlation of pivotal IRGs with the tumor microenvironment may help devise individualized immunotherapies. In our study, activated CD4+ memory T cells exhibited high infiltrating abundance in GC than normal tissue and were associated with better patient outcomes. Supporting studies have demonstrated the anti-tumor effect of activated CD4+ memory T cells in other cancers. Elevated infiltration levels of activated memory CD4 T cells are associated with increased DFS in estrogen receptor (ER)-negative or progesterone receptor (PR)-negative subtypes or combined subtypes^30^ and are closely correlated with better OS in head and neck cancer^31^. Collectively, these results emphasize the significant role of activated CD4+ T memory cells in the prognosis of different cancer types.

Here, we established an activated CD4+ memory T cells-related 3-gene prognostic model (BATF2, MYB and CD36) in gastric cancer to evaluate the activation status of CD4+ memory T cells. This model showed better prediction performance to ICB therapy than traditionally CD8+ PD-L1+ model. CD36, as a fatty acid receptor and a toll-like receptor co-receptor^32^, has been reported to contribute to epithelial-to-mesenchymal transition and metastasis of GC^33^-^38^, while its role in the TME of gastric cancer remains unclear. BATF2, the basic leucine zipper ATF-like transcription factor 2^39^, is predicted to enhance DNA-binding transcription factor activity and reverse multidrug resistance in GC cells^40^-^42^. MYB is a critical transcription factor responsible for regulating hematopoiesis. It showed great potency in T cell activation. Yao et al.^43^ found that activated CD4+ T cell infiltration was positively associated with MYB expression, especially in GC. It can also affect the differentiation and immune homeostasis of effector regulatory T cells^44^ and regulate CD8+ T cell stemness and anti-tumor response^45^. We first discovered a CD36-BATF2/MYB axis responsible for the activation of CD4+ memory T cells in GC. CD36 may serve as an upstream receptor recognizing H. pylori and fatty acid during HFD, two risk factors of GC^1^, ^45^, ^46^, and change the levels of downstream BATF2 and MYB in our study. The BATF2-MYB protein complex consequently changes the transcription of some classical T cell activation genes (IFNG, GZMB, PRF1 and TNF)^47^, ^48^, ultimately affecting the activation status of CD4+ memory T cells.

ICB is now applied as a treatment for chemo-refractory gastric cancer (cancer that has progressed after undergoing two or more lines of chemotherapy)^1^. Pembrolizumab, an inhibitor of the PD-1 receptor, was approved by the U.S. Food and Drug Administration (FDA) in 2017 for the treatment of recurrent locally advanced or metastatic gastric or gastroesophageal junction adenocarcinomas that express PD-L1^49^. In our study, we found that the TME of the high-risk group was composed of high infiltration levels of endothelial cells, fibroblasts and myeloid dendritic cells, and a lack of CD8+T cells and NK cells, creating an immunosuppressive environment unfavorable for immunotherapy. We further established a CD36\BATF2\MYB model to evaluate the prognosis. This model helped screen for more potential responders in the CD8- and PD-L1- groups and showed better potency in predicting ICB efficacy than the traditional PD-L1 and CD8 models. When the two models were combined, we found that CD8+ PD-L1+ MYB+ BATF2+ CD36- had the best response to ICB (AUC=0.821), indicating its potential clinical application in selecting patients suitable for immunotherapy.

Overall, our results indicated the prognostic value of activated CD4+ memory T cells in GC. We created a CD36-MYB-BATF2 signature to improve the prediction accuracy of the immunotherapy response. Mechanistically, we hypothesized that CD36 might repress the activation of CD4+ memory T cells by inhibiting CXCL3-BATF2/MYB axis.

## Supplementary Material

Supplementary methods, figures and tables.

## Abbreviations

IRGs: immune cell-related genes
TCGA: The Cancer Genome Atlas
GEO: Gene Expression Omnibus
K-M: Kaplan-Meier
TIME: tumor immune microenvironment
IHC: immunohistochemistry
OS: overall survival
ICB: immune checkpoint blockage
MSI: microsatellite instability
PD-L1: programmed cell death ligand 1
TIICs: immunotherapy and tumor-infiltrating immune cells
MTCs: memory T cells
FFPE: Formalin-fixed, paraffin-embedded
DEICRGs: prognostic differentially expressed IRGs
DCA: decision curve analysis
GO: Gene Ontology
KEGG: Kyoto Encyclopedia of Genes and Genomes
co-IP: co-immunoprecipitation
ELISA: enzyme-linked immunosorbent assay
qPCR: quantitative polymerase chain reaction
ANOVA: one-way analysis of variance
FDA: Food and Drug Administration
