# Supplementary material for: CD36-BATF2\MYB Axis Predicts Anti-PD-1 Immunotherapy Response in Gastric Cancer
Source: Int J Biol Sci. 2023 Aug 21;19(14):4476–92. doi: 10.7150/ijbs.87635 (PMC10535701; doi:10.7150/ijbs.87635)
Supplement: Supplementary file 1 — Supplementary methods, figures and tables. [file ijbsv19p4476s1.pdf]

# Supplementary Online Content

## CD36-BATF2\MYB Axis Predicts Anti-PD-1 Immunotherapy Response in Gastric Cancer

Qiuyu Jiang [1†], Zhixue Chen[1†], Fansheng Meng[2†], Hao Zhang[3, 4], He Chen[1], Jindan Xue[5], Xizhong shen[1], Tianshu Liu[6*], Ling Dong[1*], Si Zhang[7*], Ruyi Xue[1, 8 *]

**Supplementary Appendix.**

**Supplementary methods.**

**Fig S1. The infiltration of different immune cells in GC. A** Heatmap showing the expression level of immune cells in every sample of dataset TCGA-STAD and GSE66229. **B** Kaplan–Meier survival curves of high and low groups of different immune cells in GSE66229 datasets.

**Fig S2. Identification of gene modules related to activated CD4+ memory T cells by weighted co-expression network. A** Sample dendrogram and trait heatmap of TCGA-STAD and GSE66229 datasets. **B** Suitable soft thresholding power beta was chosen as 12 and 3 separately. **C, D** Clustering dendrograms showed the found modules (color bars below) in two datasets detected by dynamic tree cut.

**Fig S3. Independent prognosis value of our risk score system. A** Risk score distribution and survival status scatter plots of patients in two cohorts based on the prognosis model. **B-E** Forest plots of risk score and clinical parameters by univariate and multivariate Cox regression in TCGA-STAD and GSE66229 dataset.

**Fig S4. Relationship between clinical features and risk score in TCGA-STAD and GSE66229 datasets. A, B** The heatmap of correlation between risk score-related genes (CD36, BATF2, MYB) and each clinical characteristic for two datasets. **C** Boxplots illustrated the distribution of risk scores across age groups. **D** The percentages of different age population between high-risk group and low-risk group. **E** Boxplots illustrated the distribution of risk scores across groups with different clinical features. **F** The percentages of different age, stage, T and N population among different risk score groups.

**Fig S5. GO, KEGG and immune cell infiltration analysis in TCGA-STAD and GSE66229 datasets. A** Venn diagram showed 167 differentially expressed genes in high and low risk score groups. **B, C** GO and KEGG analysis suggested that the activated CD4+ memory T cell-related model might

correlate with multiple cancer-related signaling pathways. **D** Boxplots showed the differentially expressed immune cells in high and risk score groups. **E** The correlation between immune cell infiltration and risk scores. ns indicated no statistically significance, *p<0.05, **p<0.01, *** p<0.001.

**Fig S6. CD36, BATF2 and MYB may influence the activation of CD4+ memory T cells. A** Correlation analysis between 3 key genes (MYB, BATF2, CD36) and T cell CD4+ memory infiltration in the TIMER2.0 database. **B** Scatter diagram showed the correlation between 3 key genes and some classical T cell function gene signatures (T cell-inflamed GEP, T cell dysfunction, T cell exclusion) using TIGER database. **C** Chord diagram showed the correlation between 3 key genes (MYB, BATF2, CD36) and classical T cell effector molecules (IFNG, GZMB, PRF1, and TNF).

**Fig S7. MYB was a transcription factor responsible for activation of T cells and interacted with BATF2. A** Violin plots showed the expression of MYB and BATF2 in different cells in gastric cancer microenvironment. **B** Using STRING database to detect the relationship among MYB, BATF2 and CD36. **C, D** Four binding promoters of IFNG, GZMB, PRF1 and TNF with MYB in a ChIP-Seq dataset (GSM1442006) were predicted.

**Fig. S8 CD36 might be an upstream risk factor for gastric cancer. A** Violin plot of a single-cell sequencing dataset (GSE134520) showed the high expression of CD36 in malignant cells. **B, C** Boxplots revealed that in the high-fat diet and status of Helicobacter pylori infection, CD36 was highly expressed. **D** Violin plot of the same dataset showed CD36 was also highly expressed in malignant cells under the infection of Helicobacter pylori. **E** Heatmap showed the correlation between cytokines and TLR4, TLR6, CD36, BATF2, MYB and classical T cell effector molecules (IFNG, GZMB, PRF1, and TNF). ns indicated no statistically significance, *p<0.05, **p<0.01, *** p<0.001.

**Fig. S9 CD36 had no direct impact on proliferation of gastric cancer cells. A, B** CD36 overexpression or knockdown had no impact on gastric cancer cell colony formation. Relative colony numbers were counted and shown in B. **C** CD36 overexpression or knockdown had no impact on proliferation. CCK-8 assays were performed with the stable cells above. ns indicated no statistically significance.

**Table S1.** Baseline characteristics between high- and low- risk groups in GSE66229.

**Table S2.** Baseline characteristics between high- and low- risk groups in TCGA-STAD.

**Table S3.** Gene correlation analysis of CD36, MYB, BATF2, GZMB, IFNG, PRF1 and TNF.

**Table S4.** Primers used in the study.

This supplemental material has been provided by the authors to give readers additional information about their work.

## Supplementary methods

### Analysis of immune cell infiltration landscape

The proportion of immune cells in GC and normal samples in the TCGA-STAD and GSE66229 datasets was estimated using the CIBERSORT algorithm based on the LM22 immune cell gene matrix[1]. To further investigate the relationship between the proportion of immune cells and prognosis, patients with GC were divided into high- and low-abundance groups of each differential immune cell based on the optimal value of the abundance of differential immune cells calculated by the surv_cutpoint function in the survival package. The Kaplan-Meier (K-M) curve was used to compare overall survival (OS) between the two groups.

### Construction and validation of immune-score prognostic model

Univariate Cox regression analysis was performed using the survival R package to screen for prognostic differentially expressed IRGs (DEICRGs), and multivariate Cox regression analysis was performed to identify signature genes. An immune score model was established based on a linear combination of the multiplication of the Cox baseline hazard function ($h0(t)$) and regression coefficient ($\beta$) obtained by multivariate Cox and Stepwise regression analysis. Nomograms integrating selected independent prognostic factors were established, and accuracy was evaluated using decision curve analysis (DCA)
and calibration curves.

### Cell lines and T cell activation assay

Jurkat, Clone E6-1, purchased from the Chinese Academy of Sciences, were cultured in RPMI 1640 medium (Invitrogen, 11875-093) containing 10% fetal bovine serum (16140071, Gibco), 1% glutamax (Invitrogen, 35050-061), 1% sodium pyruvate 100 mM solution (Invitrogen, 11360070) and 1% penicillin/streptomycin (Sigma, 381074). ImmunoCult™ Human CD3/CD28 T Cell Activator (StemCell, 10971) and 30 ng/ml IL-2 (R&D, 202-IL-050) were used for T cell activation for 3 days[2]. AGS and HGC-27 were also purchased from the Chinese Academy of Sciences. Human CXCL3 (MCE, HY-P72678) was added at a concentration of 10 ng/ml[3] with or without SB225002 (MCE, HY-16711), a CXCR2 antagonist, at a concentration of 12.5 μM[4] for 24 h for functional assays.

**Co-Culture system**

For co-culture, Jurkat T cells were first activated using the above methods and were then directed co-cultured with gastric cancer cells (AGS or HGC-27) at a ratio of 1:10 for 24 h. After co-cultured, Jurkat T cells in the supernatant were harvested for WB and qPCR while tumor cells were harvested after digested with trypsin for cell proliferation assay.

**RNA transfection**

BATF2 was silenced by transfecting siRNAs (Genepharma, China) according to the manuscript. Briefly, $5*10^6$ cells/ml Jurkat T cells were cultured in 24-well plate for 12h before transfection. Diluting 2 μl of Lipofectamine 2000 (Thermo, 11668019) with 50 μl of Opti-MEM, and diluting 20pmol of siRNA with 50 μl of Opti-MEM. Next, two dilution were mixed and incubated for 20 min. Non-targeting siRNA was used as a negative control. The protein expression level of WYB was measured by western blot after 48 hours. Also, AGS and HGC-27 cells transduced with lentiviral vectors (Genepharma, China) overexpressing CD36 or knocking down CD36. Transduced cells were detected by GFP fluorescence.

**Western blot**

Cells were lysed in RIPA lysis buffer (Beyotime, P0013B) containing PMSF (Beyotime, ST506) to extract total protein after centrifugation and was boiled at 95℃ for 10 minutes. Western blot was performed as described previously[5]. Briefly, protein was transferred to the PVDF membrane after electrophoretic separation. The membrane was then incubated with the diluted primary antibodies: CD36 (Proteintech, 18836-1-AP, 1:1000), MYB (Bioss, bs-5978R, 1:1000), BATF2 (Proteintech, 16592-1-AP, 1:300), PRF1 (Proteintech, 14580-1-AP, 1:10000), GZMB (Abcam, ab255598, 1:1000), TNF (Proteintech, 60291-1-Ig, 1:2000), IFNG (Proteintech, 15365-1-AP, 1:6000), β-actin (Proteintech, 66009-1-Ig, 1:10000) overnight at 4℃ and 1:10000 secondary antibodies (Proteintech, SA00001-1/,SA00001-2, 1:10000) for 1 hour the next day. Chemiluminescence was performed after washed with TBST for the result.

**Immunohistochemistry**

Tissue slides (4μm thickness) were prepared from FFPE samples, deparaffinized, and rehydrated

through graded ethanol. Then, the slides were boiled in ethylenediaminetetraacetic (EDTA) (1mM, pH 8.0) buffer in a microwave oven for antigen retrieval. Then, 3% $H_2O_2$ was applied to quench endogenous peroxidase activity and the non-specific binding was blocked by incubating in blocking buffer (Beyotime, P0260). After this, the slides were incubated with the primary antibodies: CD36 (Proteintech, 18836-1-AP, 1:800), MYB (Bioss, bs-5978R, 1:200), and BATF2 (Bioss, bs-18913R, 1:200). Then, sections were incubated with indicated secondary antibodies at room temperature for 1 hour. Positive staining was visualized with DAB (3, 3-diaminobenzidine), and then counterstained with hematoxylin.

**Multiplexed immunofluorescence**

Opal 6-Plex Detection Kit (AKOYA Biosciences, NEL821001KT) was used for Multiplexed immunofluorescence, according to the manufacturer's recommendation. Primary antibodies and their fluorophores are listed as follows: CD4 (Abcam, ab213215, 1:50, Opal 520) , GZMB (Abcam, ab255598, 1:3000, Opal 650), CD45RO (Bioss, bs-1708R, 1:200, Opal 690); α-SMA (CST, 19245S, 1:800, Opal 480), CD31 (Proteintech, 11265-1-AP, 1:800, Opal 520), CD11c (Abcam, ab52632, 1:200, Opal 570), CD8 (CST, 85336S, 1:400, Opal 650), CD56 (CST, 99746T 1:200, Opal 690).

\

**Co-immunoprecipitation (co-IP)**

Cells were cultured in 100 mm dishes until they reached 90% confluence. The cells were then washed three times with cold phosphate-buffered saline (PBS) and lysed in IP lysis buffer with 1 × protease inhibitor cocktail. The lysate was incubated for 90 minutes on ice and centrifuged for 10 minutes at 12,000 rpm at 4° C. For the co-IP assay, the cell lysates were immunoprecipitated with 2μg anti-MYB antibody (Proteintech, 17800-1-AP) and normal rabbit IgG (CST, 2729S), followed by adsorption to protein A/G beads (Santa Cruz, sc-2003). The samples were incubated overnight at 4° C on a rotating rocker, washed three times with cold IP lysis buffer, and analyzed by western blotting. Five percent of input samples were used as a positive control.

**Enzyme-linked immunosorbent assay (ELISA)**

The CXCL3 protein in supernatant was measured by ELISA using the Human CXCL3 ELISA Kit (MULTI SCIENCES, EK1265-AW1), according to the manufacturer's instructions.

**Cell proliferation assay**

Cells were seeded at a density of $1 \times 10^3$ cells per well in a 96-well plate. After 24 hours of incubation, CCK8 solution (Dojindo, CK04) was added to each well, and the plate was incubated for 1 hour at 37 ° C. Absorbance at 450 nm was measured using a microplate reader (ELx800TM, BIO-TEK Instruments, Minneapolis, MN, USA). In addition, the colony formation ability of the cells was assessed by seeding $1 \times 10^3$ cells in a six-well plate and incubating for 14 days. The cells were fixed with 4% paraformaldehyde and stained with crystal violet to visualize the colonies.

Each experiment was repeated at least three times to ensure reproducibility. Statistical analysis was performed using GraphPad Prism software. Data are presented as mean ± standard deviation.

**qPCR**

Total RNA was extracted using a TRIzol reagent followed by reverse transcription into cDNA using a commercial kit. Quantitative real-time PCR was conducted on a real-time PCR instrument using SYBR master mix (Vazyme, Q711) and gene-specific primers which are listed in **Supplementary Table 4**. PCR conditions included an initial denaturation step, followed by a set of amplification cycles, and a melting curve analysis. Gene expression was calculated using the 2-△△Ct method with GAPDH as a reference gene. The experiments were performed in triplicate and the results were expressed as mean ± SD.

**Reference**

1. Newman AM, Liu CL, Green MR, Gentles AJ, Feng W, Xu Y, et al. Robust enumeration of cell subsets from tissue expression profiles. Nat Methods. 2015; 12: 453-7.

2. Hu B, Yu M, Ma X, Sun J, Liu C, Wang C, et al. IFNalpha Potentiates Anti-PD-1 Efficacy by Remodeling Glucose Metabolism in the Hepatocellular Carcinoma Microenvironment. Cancer Discov. 2022; 12: 1718-41.

3. Guan J, Weng J, Ren Q, Zhang C, Hu L, Deng W, et al. Clinical significance and biological functions of chemokine CXCL3 in head and neck squamous cell carcinoma. Biosci Rep. 2021; 41.

4. Liu YF, Liang JJ, Ng TK, Hu Z, Xu C, Chen S, et al. CXCL5/CXCR2 modulates inflammation-mediated neural repair after optic nerve injury. Exp Neurol. 2021; 341: 113711.

5. Zhou Y, Hu L, Tang W, Li D, Ma L, Liu H, et al. Hepatic NOD2 promotes hepatocarcinogenesis via a RIP2-mediated proinflammatory response and a novel nuclear autophagy-mediated DNA damage mechanism. J Hematol Oncol. 2021; 14: 9.

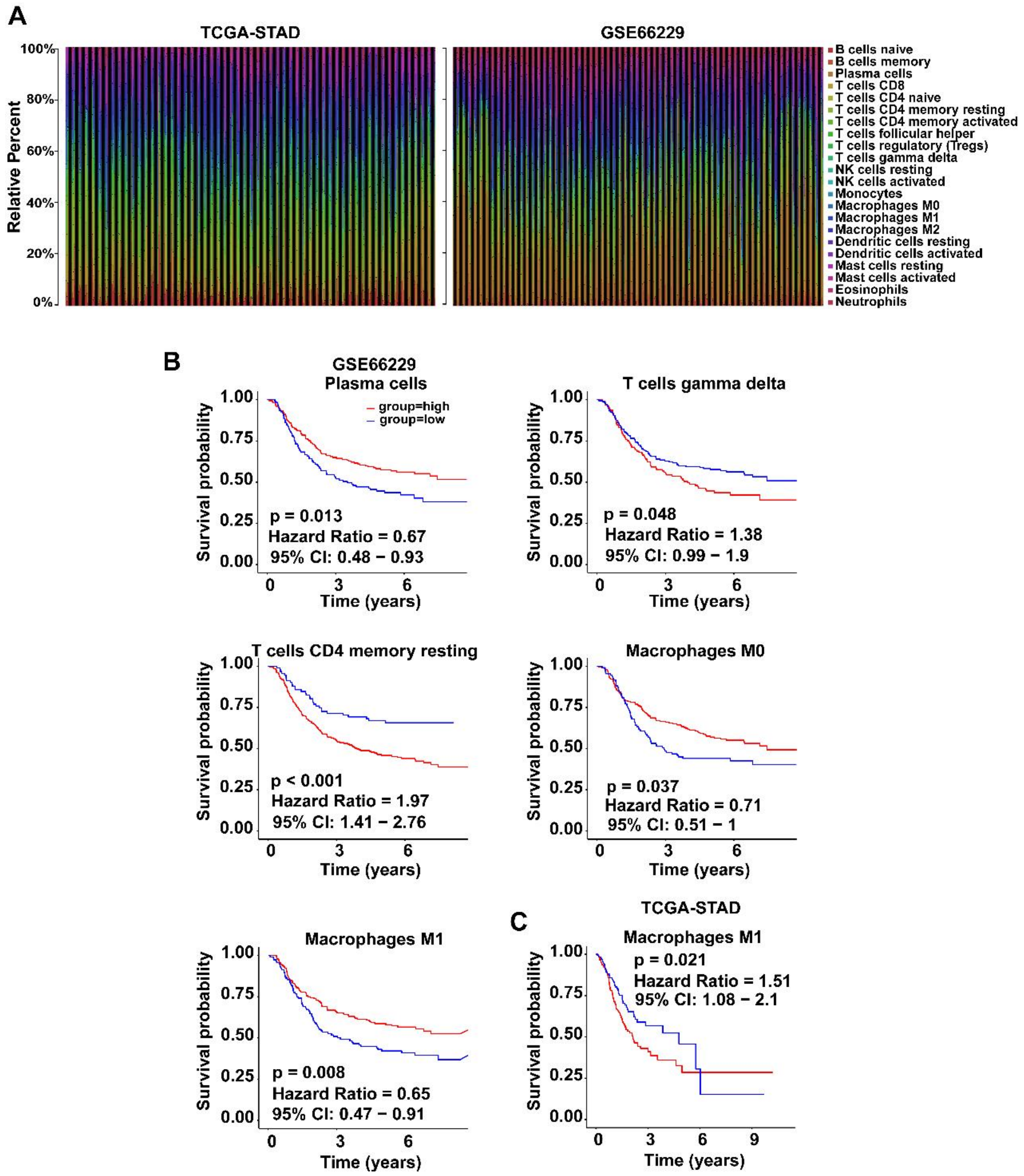


**Fig S1. The infiltration of different immune cells in GC. A** Heatmap showing the expression level of immune cells in every sample of dataset TCGA-STAD and GSE66229. **B** Kaplan–Meier survival curves of high and low groups of different immune cells in GSE66229 datasets. Survival distributions were compared using the log-rank test.

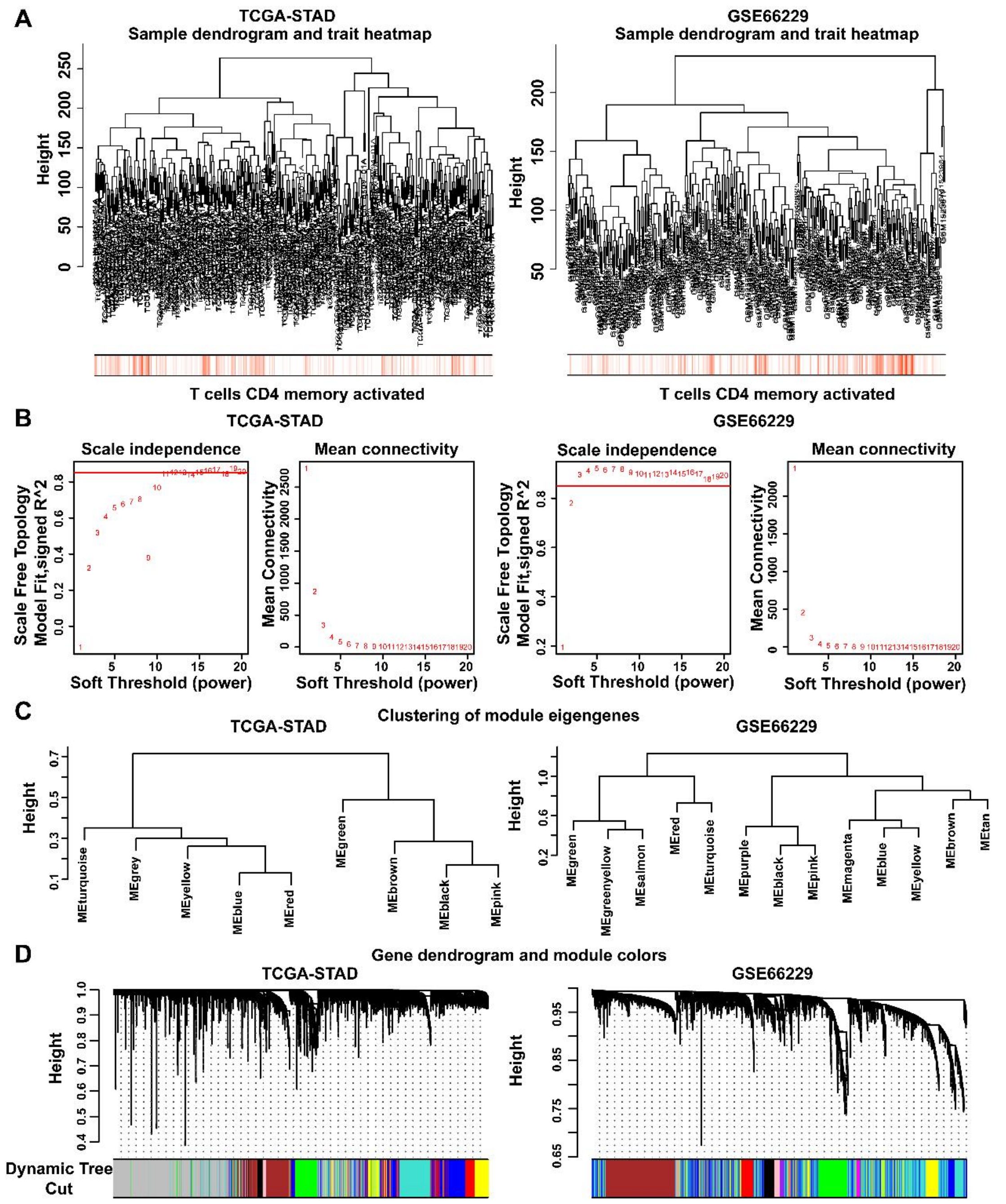


**Fig S2. Identification of gene modules related to activated CD4+ memory T cells by weighted co-expression network.** **A** Sample dendrogram and trait heatmap of TCGA-STAD and GSE66229 datasets. **B** Suitable soft thresholding power beta was chosen as 12 and 3 separately. **C, D** Clustering dendrograms showed the found modules (color bars below) in two datasets detected by dynamic tree cut.

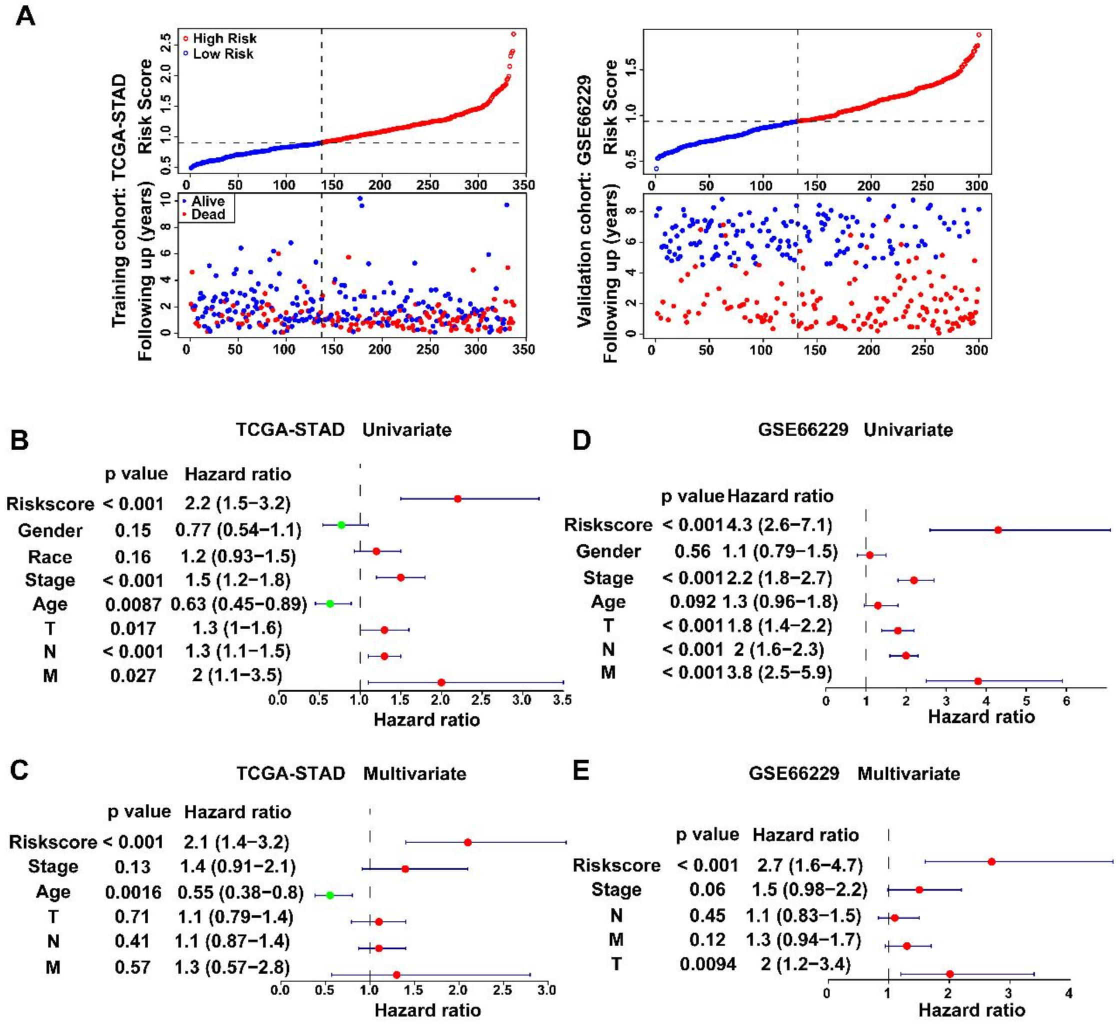


**Fig S3. Independent prognosis value of our risk score system. A** Risk score distribution and survival status scatter plots of patients in two cohorts based on the prognosis model. **B-E** Forest plots of risk score and clinical parameters by univariate and multivariate Cox regression in TCGA-STAD and GSE66229 dataset.

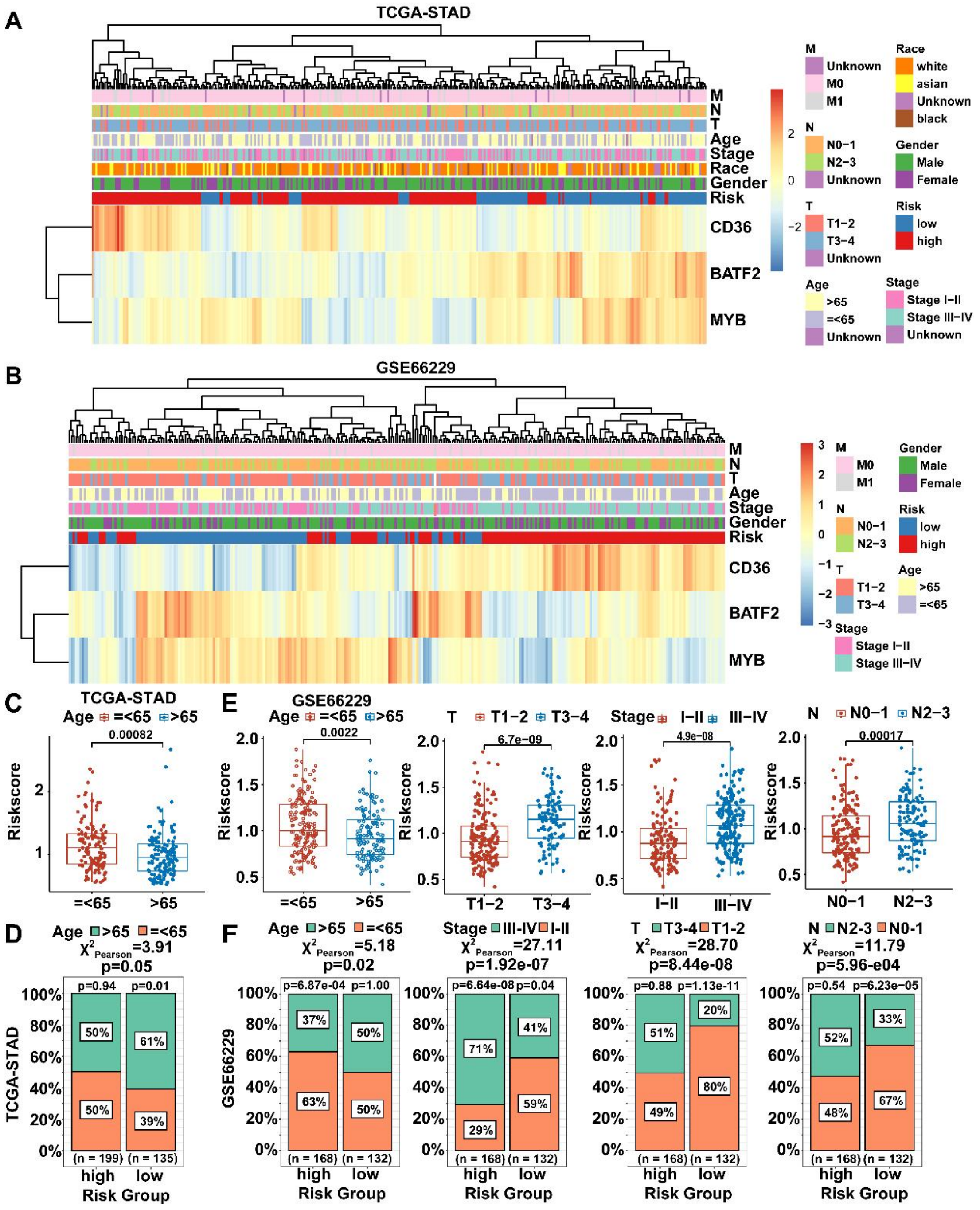


**Fig S4. Relationship between clinical features and risk score in TCGA-STAD and GSE66229 datasets. A, B** The heatmap of correlation between risk score-related genes (CD36, BATF2, MYB) and each clinical characteristic for two datasets. **C** Boxplots illustrated the distribution of risk scores across age groups. Statistical analyses: Unpaired two-tailed Student's t-test. **D** The percentages of different age population between high-risk group and low-risk group. Statistical analyses: Chi-square test. **E** Boxplots illustrated the distribution of risk scores across groups with different clinical features. Statistical analyses: Unpaired two-tailed Student's t-test. **F** The percentages of different age, stage, T and N population among different risk score groups. Statistical analyses:

Chi-square test.

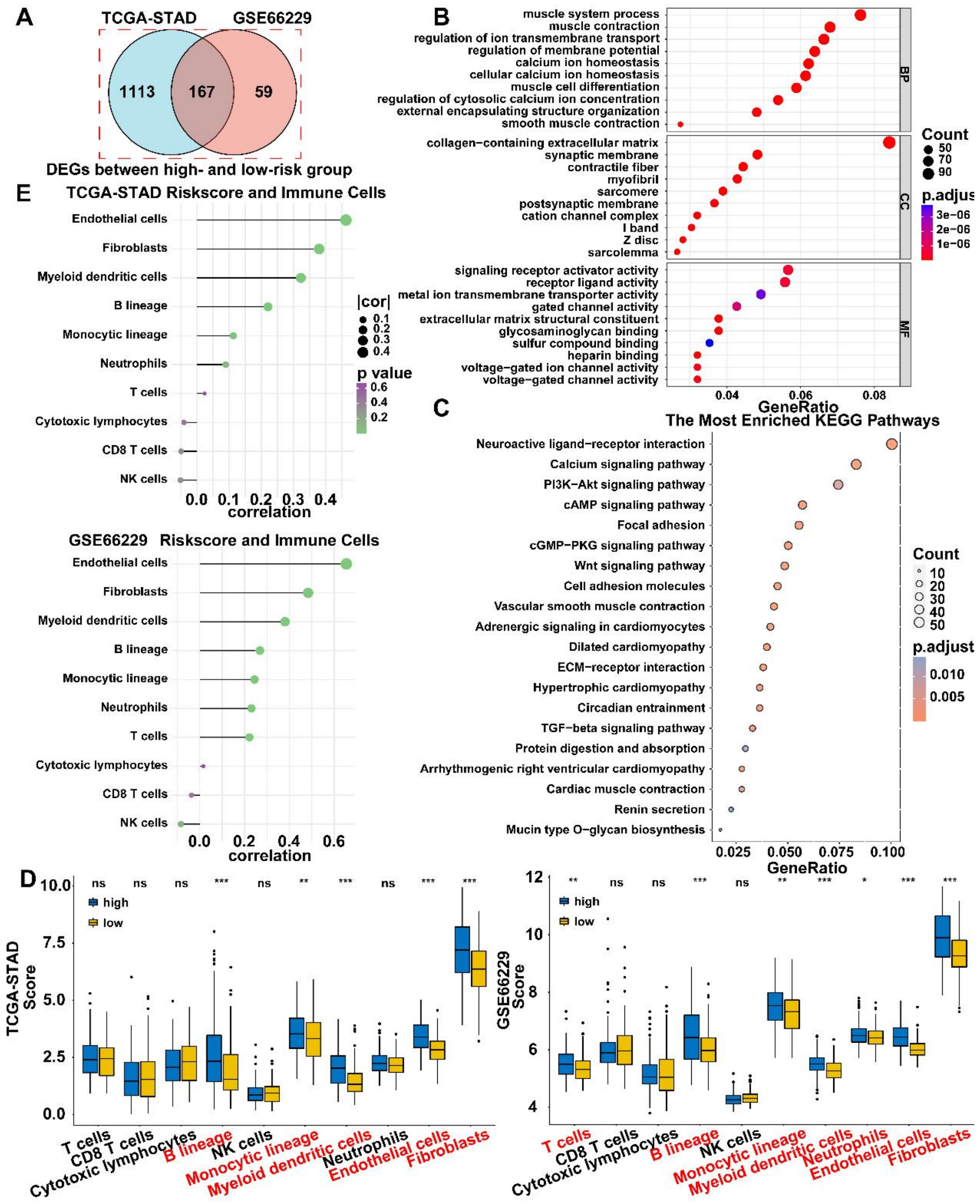


**Fig S5. GO, KEGG and immune cell infiltration analysis in TCGA-STAD and GSE66229 datasets. A** Venn diagram showed 167 differentially expressed genes in high and low risk score groups. **B, C** GO and KEGG analysis suggested that the activated CD4+ memory T cell-related model might correlate with multiple cancer-related signaling pathways. **D** Boxplots showed the differentially expressed immune cells in high and risk score groups. Statistical analyses: Unpaired two-tailed Student's t-test and or Mann Whitney test. **E** The correlation between immune cell infiltration and risk scores. Statistical analyses: Spearman correlation analysis. ns indicated no statistically significance,

*p<0.05, **p<0.01, *** p<0.001.

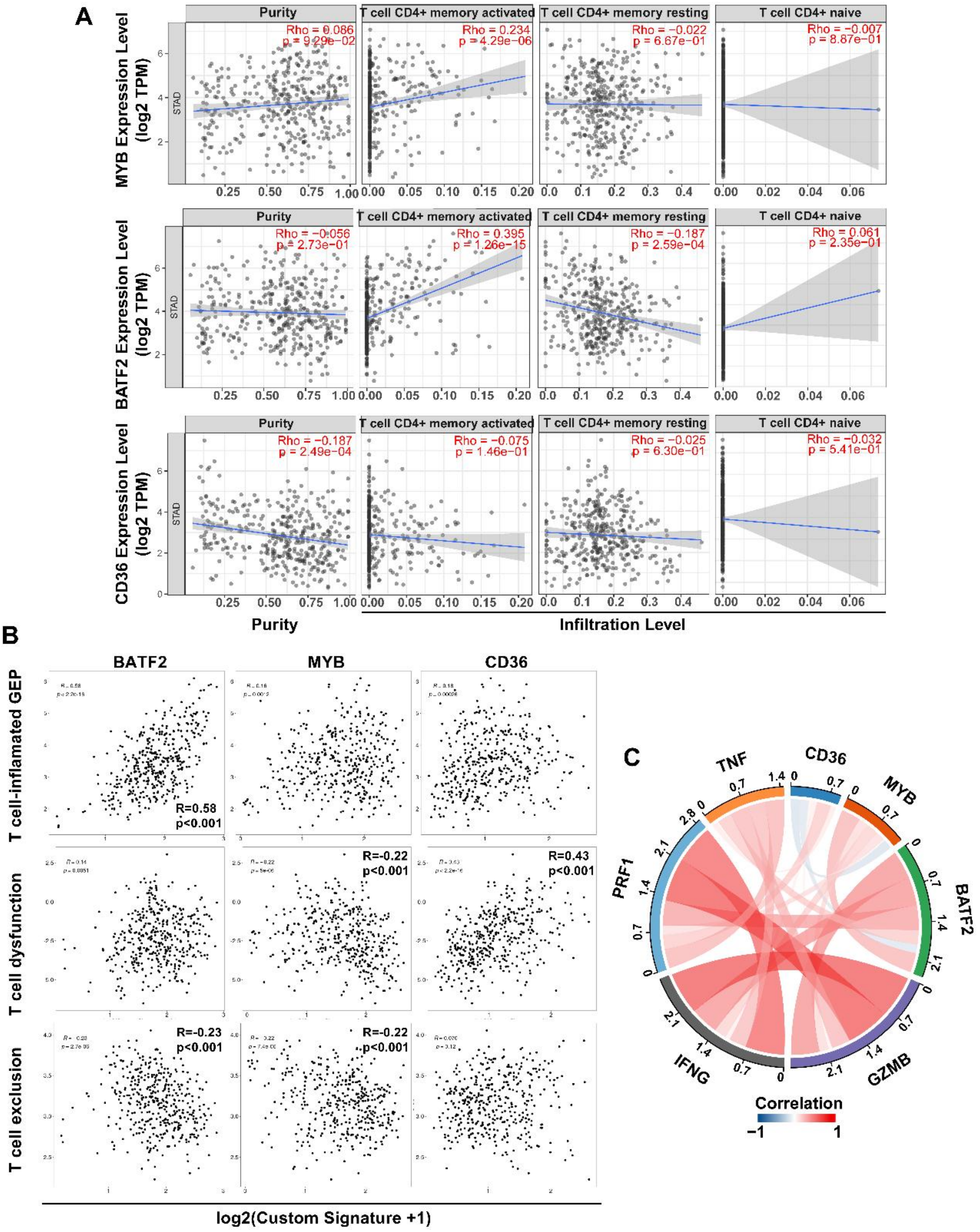


**Fig S6. CD36, BATF2 and MYB may influence the activation of CD4+ memory T cells. A** Correlation analysis between 3 key genes (MYB, BATF2, CD36) and the infiltration of T cell CD4+ memory activated, T cell CD4+ memory resting and T cell CD4+ naive in the TIMER2.0 database. Statistical analyses: Spearman correlation analysis. **B** Scatter diagram showed the correlation between 3 key genes and some classical T cell function gene signatures (T cell-inflamed GEP, T cell dysfunction, T cell exclusion) using TIGER database. Statistical analyses: Spearman correlation analysis. **C** Chord

diagram showed the correlation between 3 key genes (MYB, BATF2, CD36) and classical T cell effector molecules (IFNG, GZMB, PRF1, and TNF). Statistical analyses: Spearman correlation analysis.

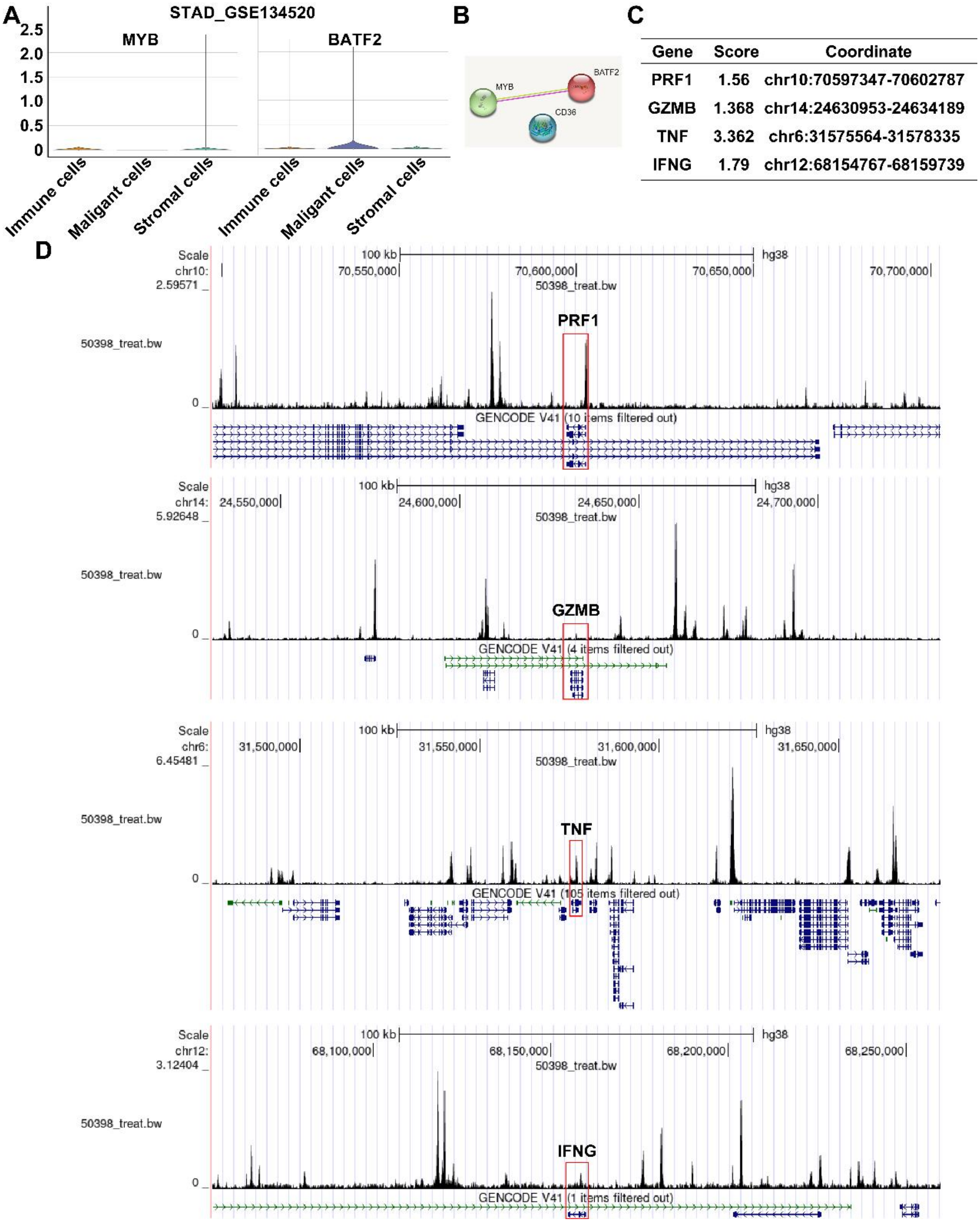


| Gene | Score | Coordinate |
|---|---|---|
| PRF1 | 1.56 | chr10:70597347-70602787 |
| GZMB | 1.368 | chr14:24630953-24634189 |
| TNF | 3.362 | chr6:31575564-31578335 |
| IFNG | 1.79 | chr12:68154767-68159739 |

**Fig S7. MYB was a transcription factor responsible for activation of T cells and interacted with BATF2. A** Violin plots showed the expression of MYB and BATF2 in different cells in gastric cancer microenvironment. **B** Using STRING database to detect the relationship among MYB, BATF2 and

CD36. **C, D** Four binding promoters of IFNG, GZMB, PRF1 and TNF with MYB in a ChIP-Seq dataset (GSM1442006) were predicted.

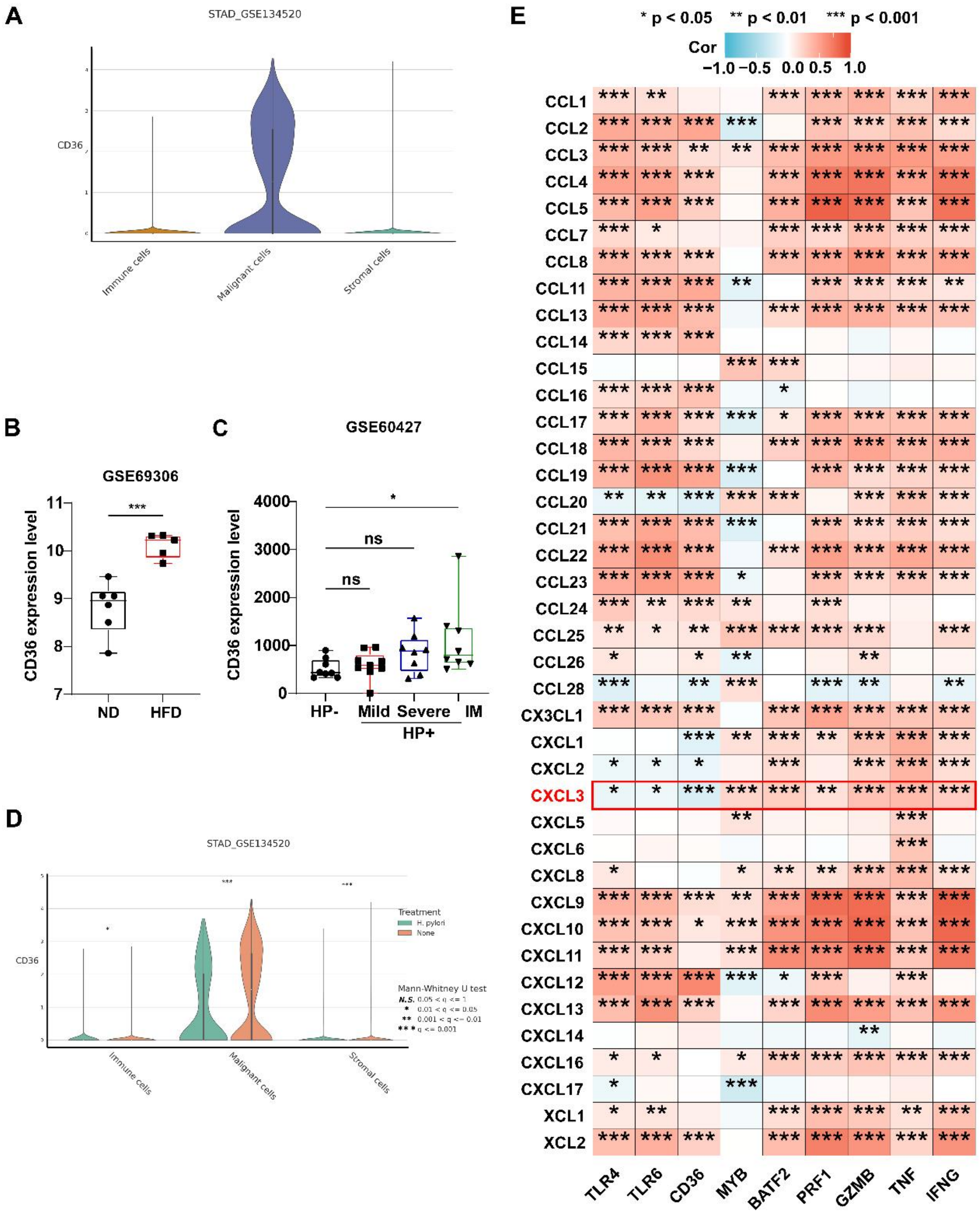


**Fig S8. CD36 might be an upstream risk factor for gastric cancer. A** Violin plot of a single-cell sequencing dataset (GSE134520) showed the high expression of CD36 in malignant cells. **B, C** Boxplots revealed that in the high-fat diet and status of Helicobacter pylori infection, CD36 was highly expressed. Statistical analyses: Unpaired two-tailed Student's t-test or One-way ANOVA and Dunnett's multiple comparisons test. **D** Violin plot of the same dataset showed CD36 was also highly expressed in malignant cells under the infection of Helicobacter pylori. Statistical analyses: Mann Whitney test.

**E** Heatmap showed the correlation between cytokines and TLR4, TLR6, CD36, BATF2, MYB and classical T cell effector molecules (IFNG, GZMB, PRF1, and TNF). Statistical analyses: Spearman correlation analysis. ns indicated no statistically significance, *p<0.05, **p<0.01, *** p<0.001.

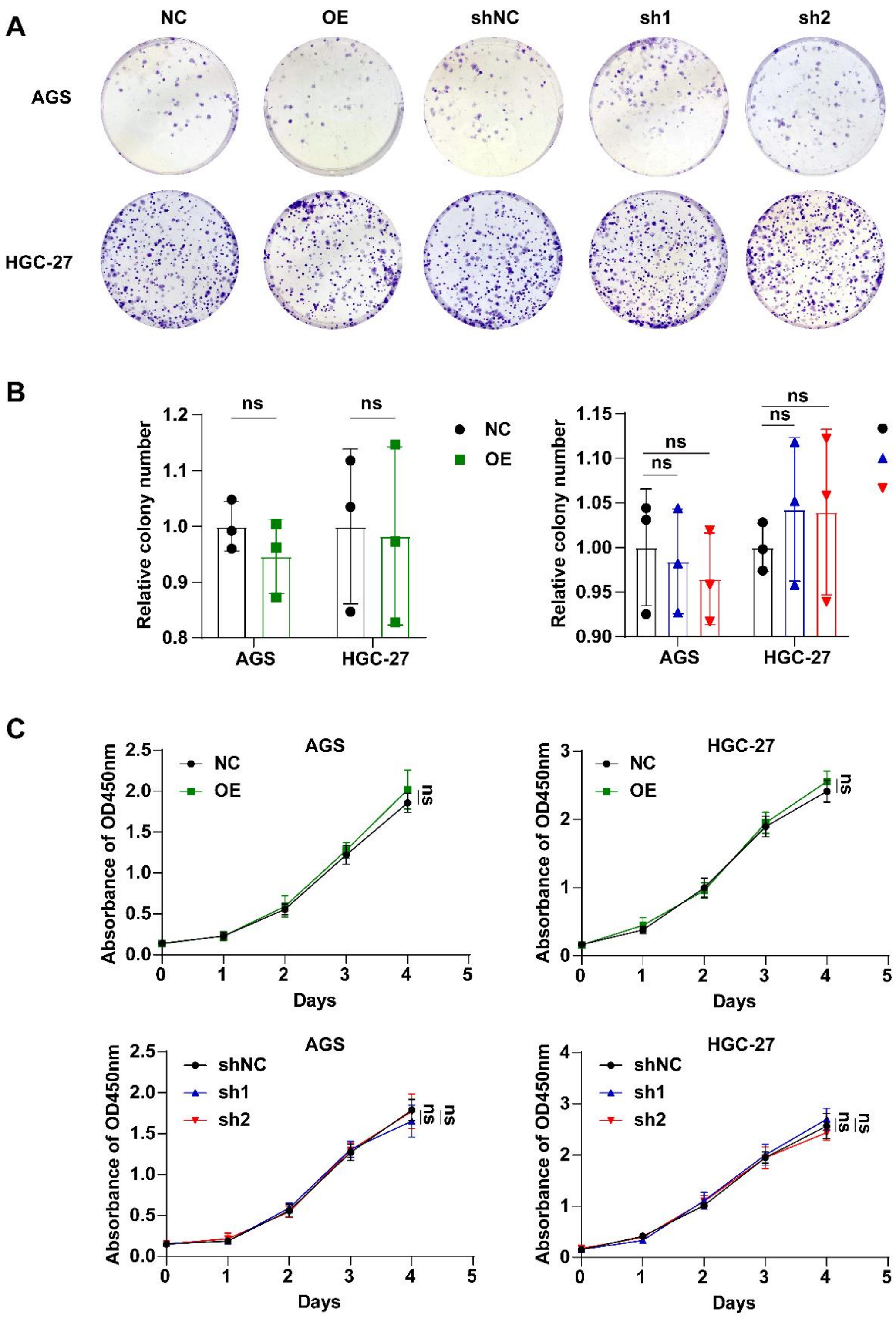


**Fig. S9 CD36 had no direct impact on proliferation of gastric cancer cells. A, B** CD36

overexpression or knockdown had no impact on gastric cancer cell colony formation. Relative colony numbers were counted and shown in B. Statistical analyses: Unpaired two-tailed Student's t-test or One-way ANOVA and Dunnett's multiple comparisons test. **C** CD36 overexpression or knockdown had no impact on proliferation. CCK-8 assays were performed with the stable cells above. ns indicated no statistically significance. Statistical analyses: Two-way ANOVA and Dunnett's multiple comparisons test, Two-way ANOVA and Šídák's multiple comparison test.

**Table S1.** Baseline characteristics between high- and low- risk groups in GSE66229.

| | | Score | | |
|---|---|---|---|---|
| | Total (N=337) | high (N=200) | low (N=137) | P-value |
| **Gender** | | | | 0.339 |
| Female | 119 (35.3%) | 66 (33.0%) | 53 (38.7%) | |
| Male | 218 (64.7%) | 134 (67.0%) | 84 (61.3%) | |
| **Age** | | | | 0.0927 |
| =<65 | 153 (45.4%) | 100 (50.0%) | 53 (38.7%) | |
| >65 | 181 (53.7%) | 99 (49.5%) | 82 (59.9%) | |
| Unknown | 3 (0.9%) | 1 (0.5%) | 2 (1.5%) | |
| **Race** | | | | 0.122 |
| asian | 68 (20.2%) | 40 (20.0%) | 28 (20.4%) | |
| black | 10 (3.0%) | 4 (2.0%) | 6 (4.4%) | |
| Unknown | 45 (13.4%) | 21 (10.5%) | 24 (17.5%) | |
| white | 214 (63.5%) | 135 (67.5%) | 79 (57.7%) | |
| **Stage** | | | | 0.219 |
| Stage I-II | 152 (45.1%) | 85 (42.5%) | 67 (48.9%) | |
| Stage III-IV | 171 (50.7%) | 104 (52.0%) | 67 (48.9%) | |
| Unknown | 14 (4.2%) | 11 (5.5%) | 3 (2.2%) | |
| **Pathologic_T** | | | | 0.235 |
| T1-2 | 89 (26.4%) | 51 (25.5%) | 38 (27.7%) | |
| T3-4 | 244 (72.4%) | 145 (72.5%) | 99 (72.3%) | |
| Unknown | 4 (1.2%) | 4 (2.0%) | 0 (0%) | |
| **Pathologic_N** | | | | 0.128 |
| N0-1 | 190 (56.4%) | 104 (52.0%) | 86 (62.8%) | |
| N2-3 | 136 (40.4%) | 88 (44.0%) | 48 (35.0%) | |
| Unknown | 11 (3.3%) | 8 (4.0%) | 3 (2.2%) | |

| | | | | |
|---|---|---|---|---|
| **Pathologic_M** | | | | 0.418 |
| M0 | 303 (89.9%) | 177 (88.5%) | 126 (92.0%) | |
| M1 | 22 (6.5%) | 16 (8.0%) | 6 (4.4%) | |
| Unknown | 12 (3.6%) | 7 (3.5%) | 5 (3.6%) | |

**Table S2.** Baseline characteristics between high- and low- risk groups in TCGA-STAD.

| | Total | Score | | P-value |
|---|---|---|---|---|
| | | high | low | |
| | (N=300) | (N=168) | (N=132) | |
| **Gender** | | | | 0.469 |
| Female | 101 (33.7%) | 60 (35.7%) | 41 (31.1%) | |
| Male | 199 (66.3%) | 108 (64.3%) | 91 (68.9%) | |
| **Age** | | | | 0.031 |
| =<65 | 172 (57.3%) | 106 (63.1%) | 66 (50.0%) | |
| >65 | 128 (42.7%) | 62 (36.9%) | 66 (50.0%) | |
| **Stage** | | | | <0.001 |
| Stage I-II | 127 (42.3%) | 49 (29.2%) | 78 (59.1%) | |
| Stage III-IV | 173 (57.7%) | 119 (70.8%) | 54 (40.9%) | |
| **Pathologic_T** | | | | <0.001 |
| T1-2 | 188 (62.7%) | 83 (49.4%) | 105 (79.5%) | |
| T3-4 | 112 (37.3%) | 85 (50.6%) | 27 (20.5%) | |
| **Pathologic_N** | | | | <0.001 |
| N0-1 | 169 (56.3%) | 80 (47.6%) | 89 (67.4%) | |
| N2-3 | 131 (43.7%) | 88 (52.4%) | 43 (32.6%) | |
| **Pathologic_M** | | | | 0.333 |
| M0 | 273 (91.0%) | 150 (89.3%) | 123 (93.2%) | |
| M1 | 27 (9.0%) | 18 (10.7%) | 9 (6.8%) | |

**Table S3.** Gene correlation analysis of CD36, MYB, BATF2, GZMB, IFNG, PRF1 and TNF.

| Gene1 | Gene2 | Cor_spearman | p_spearman |
|---|---|---|---|
| CD36 | MYB | -0.180655137 | 0.00043866 |
| CD36 | BATF2 | -0.165058824 | 0.00133797 |
| MYB | BATF2 | 0.373757196 | 0.207122103 |
| CD36 | GZMB | 0.06529298 | 0.086178433 |
| MYB | GZMB | 0.180162476 | 6.72517E-08 |
| BATF2 | GZMB | 0.546778246 | 0.044596775 |
| CD36 | IFNG | 0.088730609 | 0.00043866 |
| MYB | IFNG | 0.23422506 | 7.06102E-14 |
| BATF2 | IFNG | 0.531751724 | 0.000455043 |
| GZMB | IFNG | 0.817448293 | 4.54996E-06 |
| CD36 | PRF1 | 0.274320401 | 0.0005341 |
| MYB | PRF1 | 0.177994994 | 0.672352274 |
| BATF2 | PRF1 | 0.478912049 | 0.00133797 |
| GZMB | PRF1 | 0.82229901 | 7.06102E-14 |
| IFNG | PRF1 | 0.752137345 | 0 |
| CD36 | TNF | 0.103781545 | 0 |
| MYB | TNF | 0.021910115 | 0 |
| BATF2 | TNF | 0.272577313 | 8.19886E-08 |
| GZMB | TNF | 0.358589601 | 0.207122103 |
| IFNG | TNF | 0.331660018 | 0.000455043 |
| PRF1 | TNF | 0.370405962 | 0 |
| CD36 | MYB | -0.180655137 | 0 |
| CD36 | BATF2 | -0.165058824 | 0 |
| MYB | BATF2 | 0.373757196 | 8.04468E-13 |
| CD36 | GZMB | 0.06529298 | 0.086178433 |
| MYB | GZMB | 0.180162476 | 4.54996E-06 |
| BATF2 | GZMB | 0.546778246 | 0 |

| | | | |
|---|---|---|---|
| CD36 | IFNG | 0.088730609 | 0 |
| MYB | IFNG | 0.23422506 | 0 |
| BATF2 | IFNG | 0.531751724 | 4.44194E-11 |
| GZMB | IFNG | 0.817448293 | 6.72517E-08 |
| CD36 | PRF1 | 0.274320401 | 0.0005341 |
| MYB | PRF1 | 0.177994994 | 0 |
| BATF2 | PRF1 | 0.478912049 | 0 |
| GZMB | PRF1 | 0.82229901 | 0 |
| IFNG | PRF1 | 0.752137345 | 1.22125E-13 |
| CD36 | TNF | 0.103781545 | 0.044596775 |
| MYB | TNF | 0.021910115 | 0.672352274 |
| BATF2 | TNF | 0.272577313 | 8.19886E-08 |
| GZMB | TNF | 0.358589601 | 8.04468E-13 |
| IFNG | TNF | 0.331660018 | 4.44194E-11 |
| PRF1 | TNF | 0.370405962 | 1.22125E-13 |

**Table S4.** Primers used in this study.

| Name | Sequences（5’-3’） |
|---|---|
| CXCL3-F | CGCCCAAACCGAAGTCATAG |
| CXCL3-R | GCTCCCCTTGTTCAGTATCTTTT |
| GAPDH-F | ACAACTTTGGTATCGTGGAAGG |
| GAPDH-R | GCCATCACGCCACAGTTTC |
| TNF-F | CCTCTCTCTAATCAGCCCTCTG |
| TNF-R | GAGGACCTGGGAGTAGATGAG |
| IFNG-F | TCGGTAACTGACTTGAATGTCCA |
| IFNG-R | TCGCTTCCCTGTTTTAGCTGC |
| GZMB-F | TACCATTGAGTTGTGCGTGGG |
| GZMB-R | GCCATTGTTTCGTCCATAGGAGA |
| PRF1-F | GTGGGACAATAACAACCCCAT |
| PRF1-R | TGGCATGATAGCGGAATTTTAGG |
